# Supplementary material for: Identification of covalent modifications regulating immune signaling complex composition and phenotype
Source: Mol Syst Biol. 2021 Jul 28;17(7):e10125. doi: 10.15252/msb.202010125 (PMC8447602; doi:10.15252/msb.202010125)
Supplement: Supplementary file 2 — Appendix [file MSB-17-e10125-s003.pdf]

Appendix

Table of content

Appendix Figure S1: Interactions and PTMs of AKT1 in Human Monocytes

Appendix Figure S2: Interactions and PTMs of BCL10 in Human Monocytes

Appendix Figure S3: Interactions and PTMs of BIRC2 in Human Monocytes

Appendix Figure S4: Interactions and PTMs of CASP4, CASP5 in Human Monocytes

Appendix Figure S5: Interactions and PTMs of CASP7 in Human Monocytes

Appendix Figure S6: Interactions and PTMs of IFIH1 in Human Monocytes

Appendix Figure S7: Interactions and PTMs of IKBKE, TRAF6 in Human Monocytes

Appendix Figure S8: Interactions and PTMs of MAP3K8 in Human Monocytes

Appendix Figure S9: Interactions and PTMs of MYD88 in Human Monocytes

Appendix Figure S10: Interactions and PTMs of RIPK2 in Human Monocytes

Appendix Figure S11: Interactions and PTMs of RIPK3 in Human Monocytes

Appendix Figure S12: Interactions and PTMs of SYK in Human Monocytes

Appendix Figure S13: Interactions and PTMs of TIRAP in Human Monocytes

Appendix Figure S14: Interactions and PTMs of TRAF1 in Human Monocytes

Appendix Table S1: Oligonucleotides for pLOC cloning

Appendix Table S2: Oligonucleotides for site-directed mutagenesis of TRAF2

Appendix Table S3: Oligonucleotides for CRISPR Cas9 gene knockout

Appendix Table S4: Oligonucleotides for epitope tagging of MAPK14 with STREP-tag

Appendix Table S5: Recombinant DNA from Precision Lenti ORF Collection, GE Healthcare

Appendix Table S6: Recombinant DNA by Gene synthesis

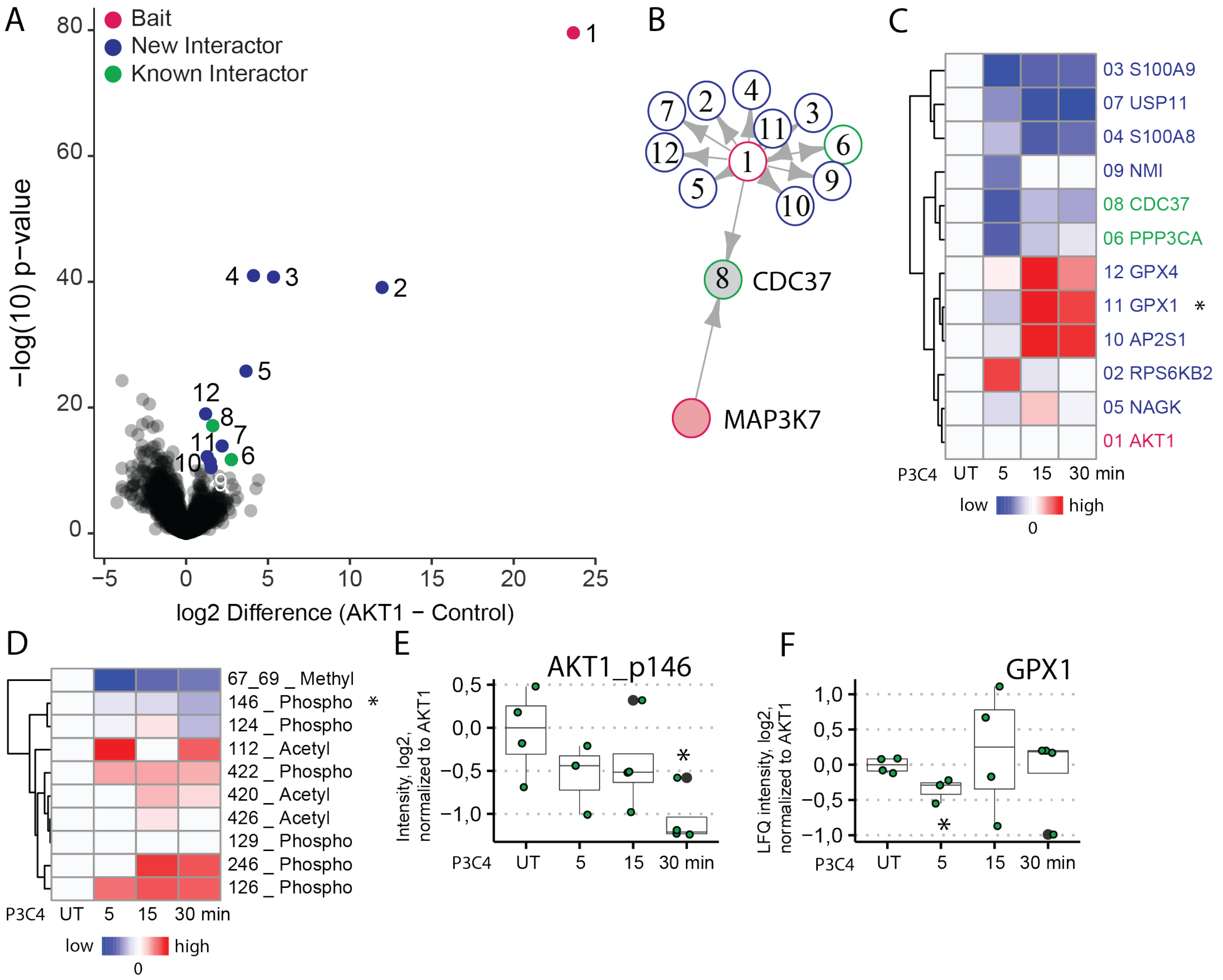

Appendix Figure S1. Related to [Fig. 4, Table 1,2,3]: Interactions and PTMs of AKT1 in Human Monocytes. (A) Volcano plot representing the interactome of AKT1 (measured 15x in biological replicates) compared against all other pull-downs in the control group. Significant interactors of AKT1 (two-tailed t-test, FDR < 0.01, enrichment >4) are colored in blue (novel interactors) and green (known interactors). (B) Interactors of TRAF2 (blue: novel interactors, green: known interactors) with interconnecting proteins between different baits colored in grey. (C) Heatmap of significant interactors of AKT1 upon TLR2 activation of human monocytes, with significant hits (two-tailed *t*-test, p-value < 0.05) denoted with an asterisk. (D) Heatmap of AKT1 PTMs (acetylation, methylation, and phosphorylation) upon TLR2 activation of human monocytes, with significant hits in at least one time-point (two-tailed *t*-test, p-value < 0.05) denoted with an asterisk. Cell activation was performed for 5, 15 and 30 min with the TLR2 ligand PAM3CSK4 (P3C4), UT was harvested after 30 min without Pam3CSK4 activation. (E) Intensity profile (normalized to AKT1 protein intensity and untreated) of the phosphorylation of AKT1 Thr146 upon TLR2 activation of human monocytes. (F) LFQ Intensity profile (normalized to AKT1 LFQ intensity and untreated) of AKT1 interactor GPX1 upon TLR2 activation of human monocytes (***: p-value < 0.001, **: p-value < 0.01, *: p-value < 0.05).

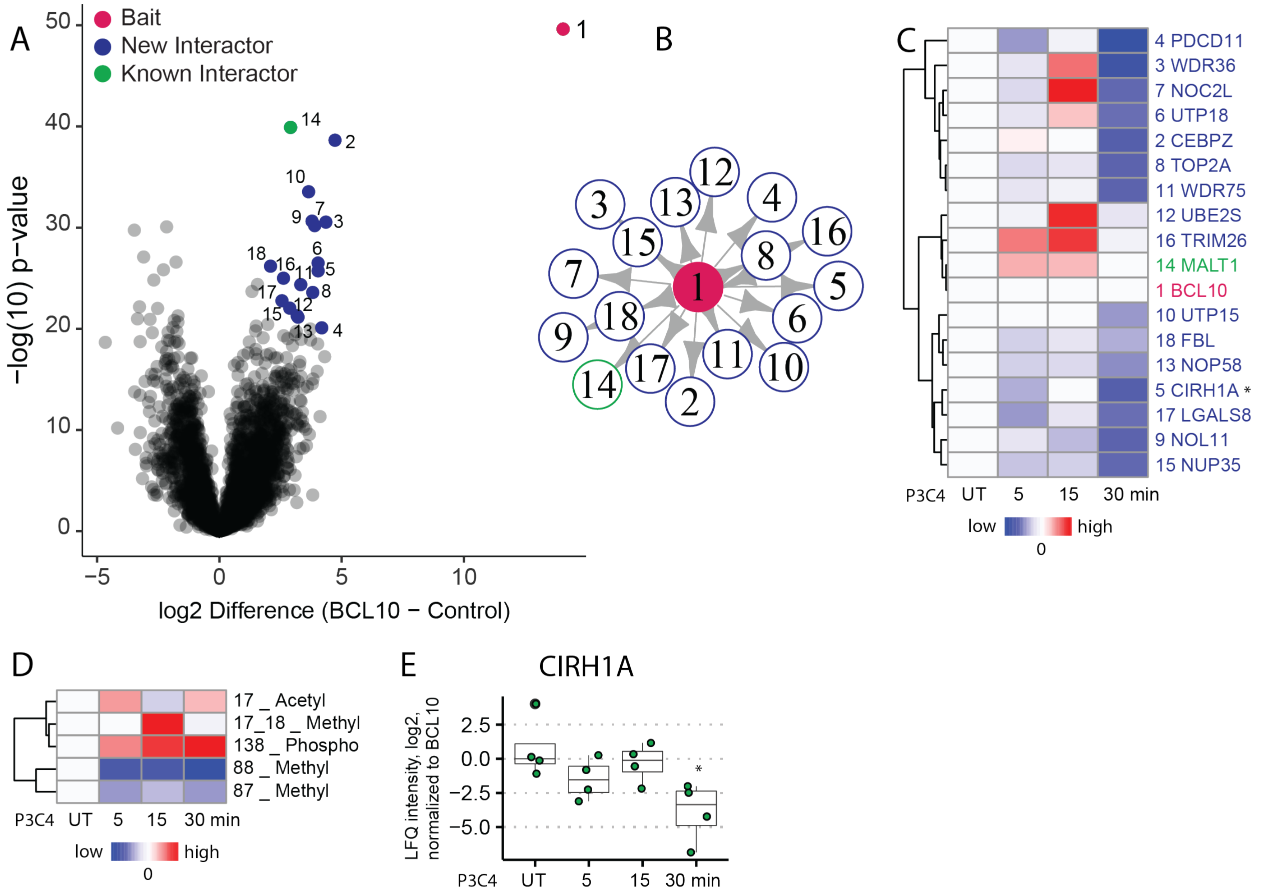

Appendix Figure S2. Related to [Fig. 4, Table 1,2,3]: Interactions and PTMs of BCL10 in Human Monocytes. (A) Volcano plot representing the interactome of BCL10 (measured 16x in biological replicates) compared against all other pull-downs in the control group. Significant interactors of BCL10 (two-tailed t-test, FDR < 0.01, enrichment >4) are colored in blue (novel interactors) and green (known interactors). (B) Interactors of TRAF2 (blue: novel interactors, green: known interactors) with interconnecting proteins between different baits colored in grey. (C) Heatmap of significant interactors of BCL10 upon TLR2 activation of human monocytes, with significant hits (two-tailed *t*-test, p-value < 0.05) denoted with an asterisk. (D) Heatmap of BCL10 PTMs (acetylation, methylation, and phosphorylation) upon TLR2 activation of human monocytes, with significant hits in at least one time-point (two-tailed *t*-test, p-value < 0.05) denoted with an asterisk. Cell activation was performed for 5, 15 and 30 min with the TLR2 ligand PAM3CSK4 (P3C4), UT was harvested after 30 min without Pam3CSK4 activation. (E) LFQ Intensity profile (normalized to BCL10 LFQ intensity and untreated) of BCL10 interactor CIRH1A upon TLR2 activation of human monocytes (***: p-value < 0.001, **: p-value < 0.01, *: p-value < 0.05).

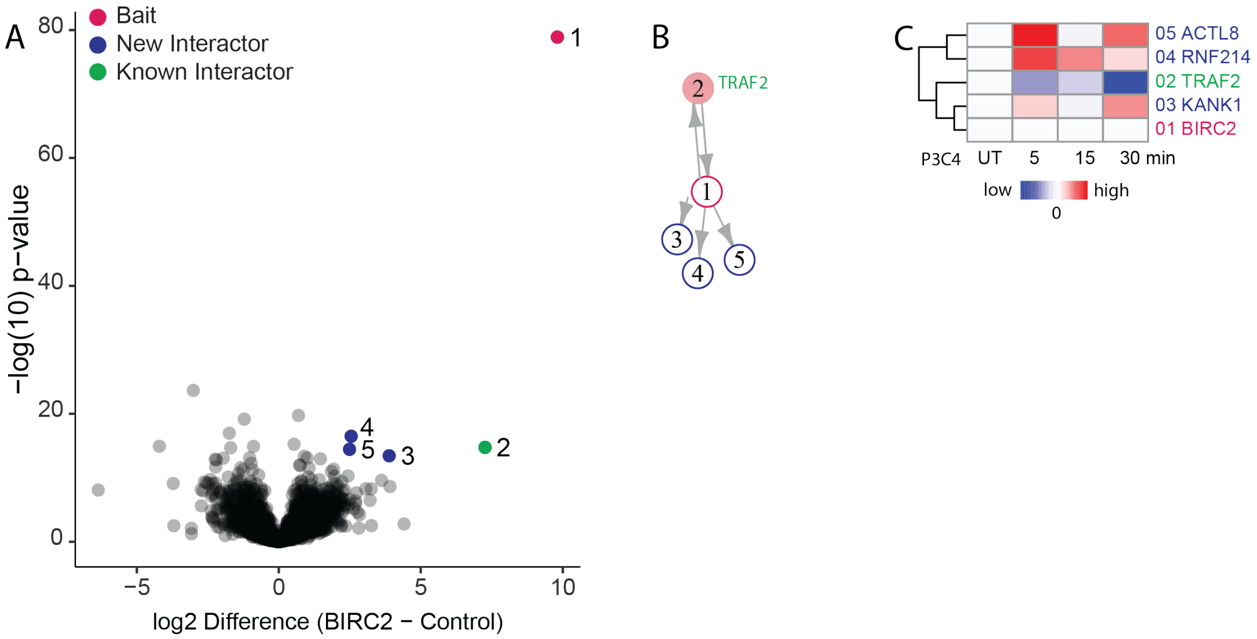

Appendix Figure S3. Related to [Fig. 4, Table 1,2,3]: Interactions and PTMs of BIRC2 in Human Monocytes. (A) Volcano plot representing the interactome of BIRC2 (measured 16x in biological replicates) compared against all other pull-downs in the control group. Significant interactors of BIRC2 (two-tailed t-test, FDR < 0.01, enrichment >4) are colored in blue (novel interactors) and green (known interactors). (B) Interactors of TRAF2 (blue: novel interactors, green: known interactors) with interconnecting proteins between different baits colored in grey. (C) Heatmap of significant interactors of BIRC2 upon TLR2 activation of human monocytes, with significant hits (two-tailed *t*-test, p-value < 0.05) denoted with an asterisk. Cell activation was performed for 5, 15 and 30 min with the TLR2 ligand PAM3CSK4 (P3C4), UT was harvested after 30 min without Pam3CSK4 activation. ***: p-value < 0.001, **: p-value < 0.01, *: p-value < 0.05).

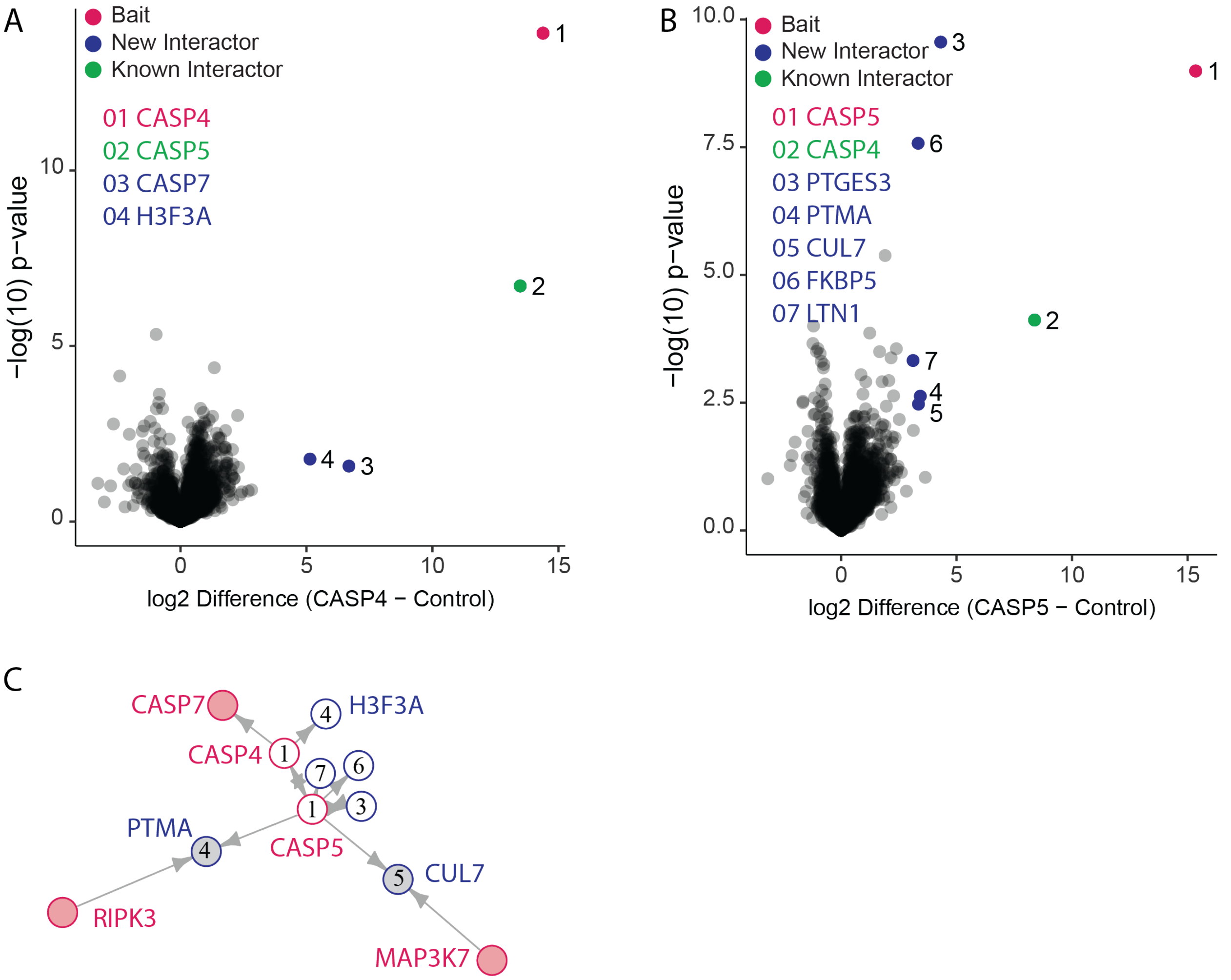

Appendix Figure S4. Related to [Fig. 4, Table 1,2,3]: Interactions of CASP4 and CASP5 in Human Monocytes. A) Volcano plot representing the interactome of CASP4 (measured 4x in biological replicates) compared against all other pull-downs in the control group. Significant interactors of CASP4 (two-tailed t-test, FDR < 0.01, enrichment >4) are colored in blue (novel interactors) and green (known interactors). B) Volcano plot representing the interactome of CASP5 (measured 4x in biological replicates) compared against all other pull-downs in the control group. Significant interactors of CASP5 (two-tailed t-test, FDR < 0.01, enrichment >4) are colored in blue (novel interactors) and green (known interactors). (C) Interactors of CASP4 and CASP5 (blue: novel interactors, green: known interactors) with interconnecting proteins between different baits colored in grey.

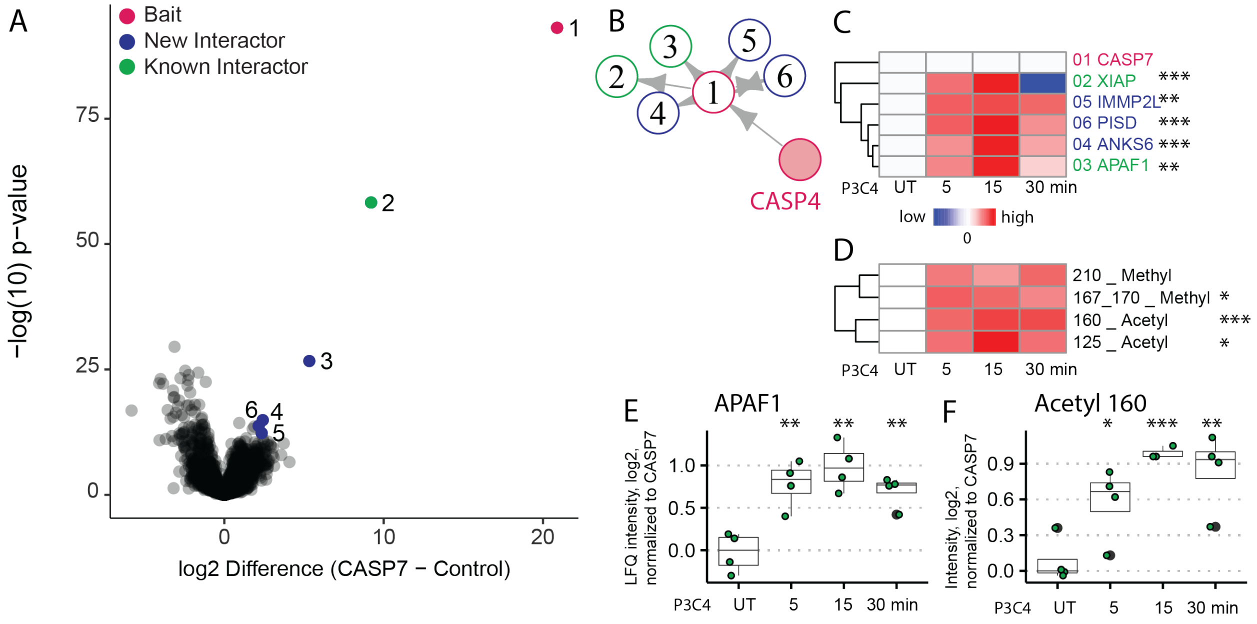

Appendix Figure S5. Related to [Fig. 4, Table 1,2,3]: Interactions and PTMs of CASP7 in Human Monocytes. (A) Volcano plot representing the interactome of CASP7 (measured 16x in biological replicates) compared against all other pull-downs in the control group. Significant interactors of CASP7 (two-tailed t-test, FDR < 0.01, enrichment >4) are colored in blue (novel interactors) and green (known interactors). (B) Interactors of CASP7 (blue: novel interactors, green: known interactors) with interconnecting proteins between different baits colored in grey. (C) Heatmap of significant interactors of CASP7 upon TLR2 activation of human monocytes, with significant hits (two-tailed t-test, p-value < 0.05) denoted with an asterisk. Cell activation was performed for 5, 15 and 30 min with the TLR2 ligand PAM3CSK4 (P3C4), UT was harvested after 30 min without Pam3CSK4 activation. (D) Heatmap of CASP7 PTMs (acetylation, methylation, and phosphorylation) upon TLR2 activation of human monocytes, with significant hits in at least one time-point (two-tailed t-test, p-value < 0.05) denoted with an asterisk. Cell activation was performed for 5, 15 and 30 min with the TLR2 ligand PAM3CSK4 (P3C4), UT was harvested after 30 min without Pam3CSK4 activation. (E) LFQ Intensity profile (normalized to CASP7 LFQ intensity and untreated) of CASP7 interactor APAF1 upon TLR2 activation of human monocytes. (E) Intensity profile (normalized to CASP7 Intensity and untreated) of CASP7 acetylation site K160 upon TLR2 activation of human monocytes. ***: p-value < 0.001, **: p-value < 0.01, *: p-value < 0.05).

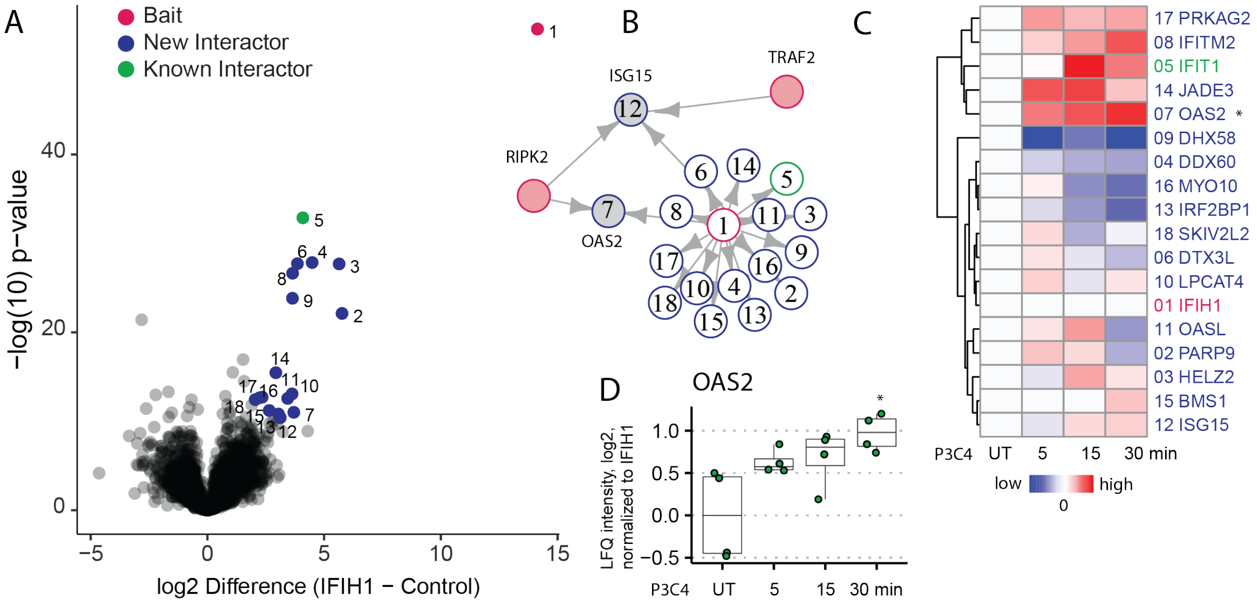

Appendix Figure S6. Related to [Fig. 4, Table 1,2,3]: Interactions and PTMs of IFIH1 in Human Monocytes. (A) Volcano plot representing the interactome of IFIH1 (measured 16x in biological replicates) compared against all other pull-downs in the control group. Significant interactors of IFIH1 (two-tailed t-test, FDR < 0.01, enrichment >4) are colored in blue (novel interactors) and green (known interactors). (B) Interactors of IFIH1 (blue: novel interactors, green: known interactors) with interconnecting proteins between different baits colored in grey. (C) Heatmap of significant interactors of IFIH1 (FDR < 0.01, enrichment >4) upon TLR2 activation of human monocytes, with significant hits (two-tailed *t*-test, p-value < 0.05) denoted with an asterisk. Cell activation was performed for 5, 15 and 30 min with the TLR2 ligand PAM3CSK4 (P3C4), UT was harvested after 30 min without Pam3CSK4 activation. (D) LFQ Intensity profile (normalized to IFIH1 LFQ intensity and untreated) of IFIH1 interactor OAF2 upon TLR2 activation of human monocytes. ***: p-value < 0.001, **: p-value < 0.01, *: p-value < 0.05).

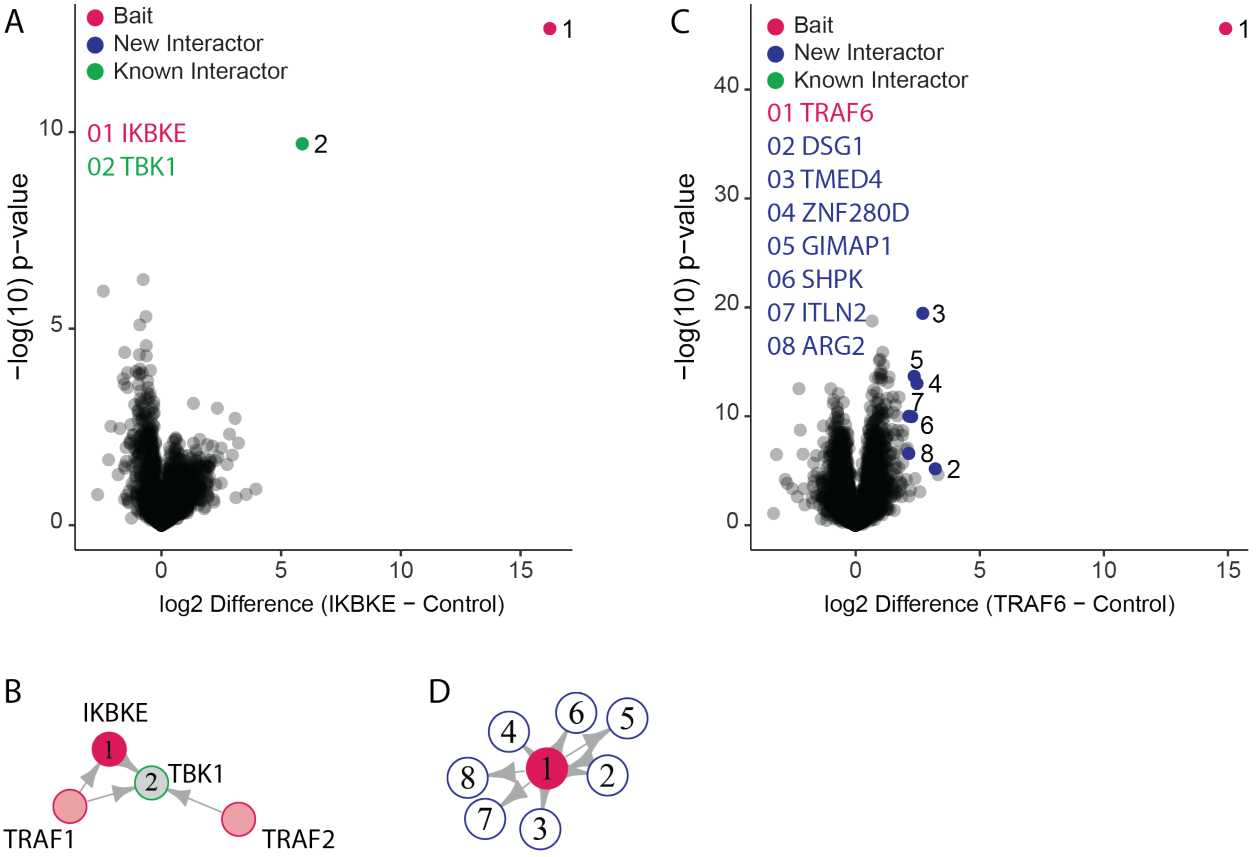

Appendix Figure S7. Related to [Fig. 4, Table 1,2,3]: Interactions of IKBKE and TRAF6 in Human Monocytes. A) Volcano plot representing the interactome of IKBKE (measured 4x in biological replicates) compared against all other pull-downs in the control group. Significant interactors of IKBKE (two-tailed t-test, FDR < 0.01, enrichment >4) are colored in blue (novel interactors) and green (known interactors). (B) Interactors of IKBKE (blue: novel interactors, green: known interactors) with interconnecting proteins between different baits colored in grey. (C) Volcano plot representing the interactome of TRAF6 (measured 16x in biological replicates) compared against all other pull-downs in the control group. Significant interactors of TRAF6 (two-tailed t-test, FDR < 0.01, enrichment >4) are colored in blue (novel interactors) and green (known interactors). (D) Interactors of TRAF6 (blue: novel interactors, green: known interactors) with interconnecting proteins between different baits colored in grey.

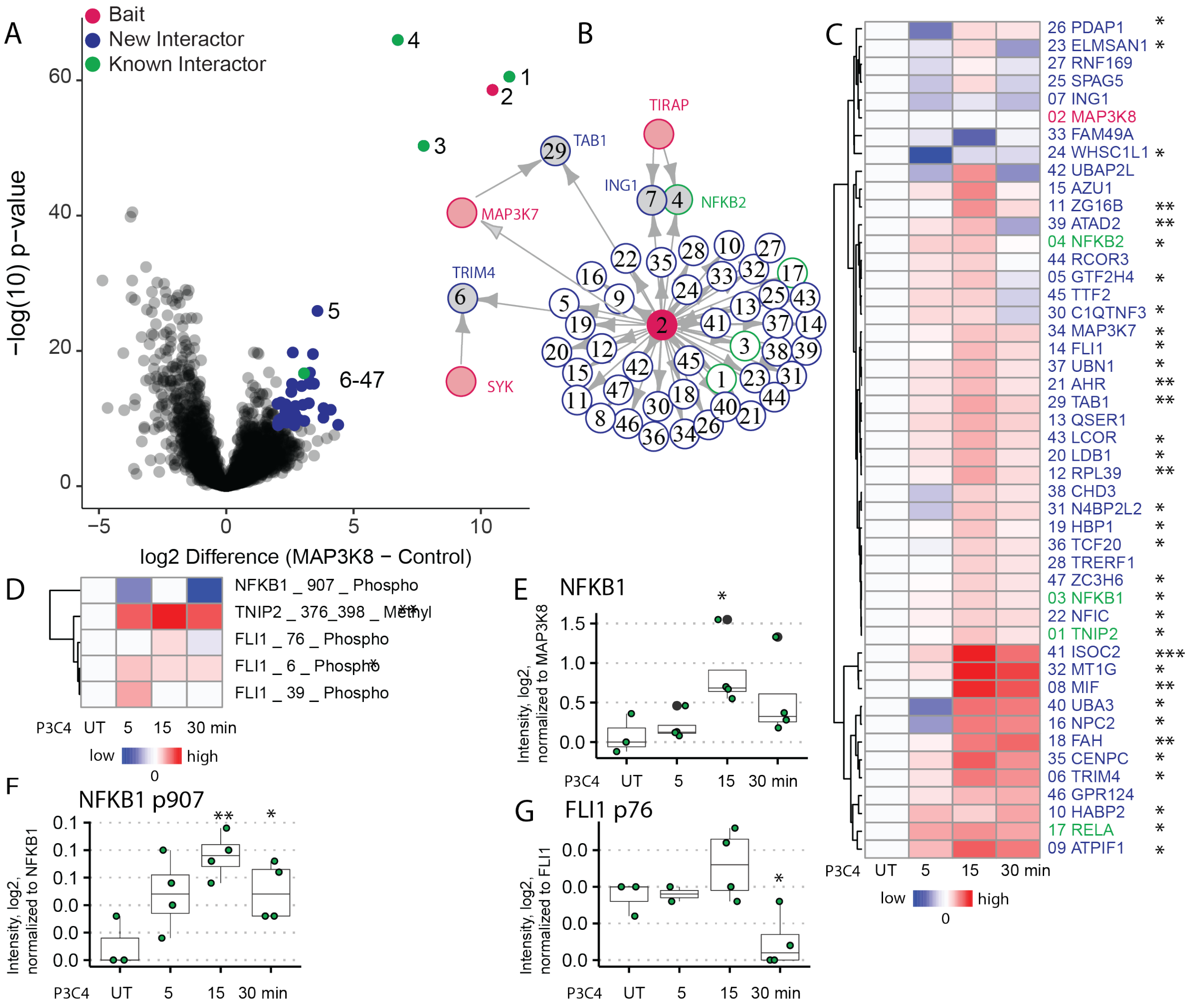

Appendix Figure S8. Related to [Fig. 4, Table 1,2,3]: Interactions and PTMs of MAP3K8 in Human Monocytes. A) Volcano plot representing the interactome of MAP3K8 (measured 15x in biological replicates) compared against all other pull-downs in the control group. Significant interactors of MAP3K8 (two-tailed t-test, FDR < 0.01, enrichment >4) are colored in blue (novel interactors) and green (known interactors). (B) Interactors of MAP3K8 (blue: novel interactors, green: known interactors) with interconnecting proteins between different baits colored in grey. (C) Heatmap of significant interactors of MAP3K8 upon TLR2 activation of human monocytes, with significant hits (two-sided *t*-test, p-value < 0.05) denoted with an asterisk. Cell activation was performed for 5, 15 and 30 min with the TLR2 ligand PAM3CSK4 (P3C4), UT was harvested after 30 min without Pam3CSK4 activation. (D) Heatmap of MAP3K8 PTMs (acetylation, methylation, and phosphorylation) upon TLR2 activation of human monocytes, with significant hits (two-sided *t*-test, p-value < 0.05) denoted with an asterisk. (E) LFQ Intensity profile of MAP3K8 interactor NFκB1 upon TLR2 activation of human monocytes, normalized to MAP3K8 bait LFQ intensity and untreated condition. (F) Intensity profile of phosphorylation of NFκB1 Ser907 upon TLR2 activation of human monocytes, normalized to NFκB1 intensity and untreated condition. (G) Intensity profile of the phosphorylation of FLI1 Ser76 upon TLR2 activation of human monocytes, normalized to FLI1 intensity and untreated condition. ***: p-value < 0.001, **: p-value < 0.01, *: p-value < 0.05).

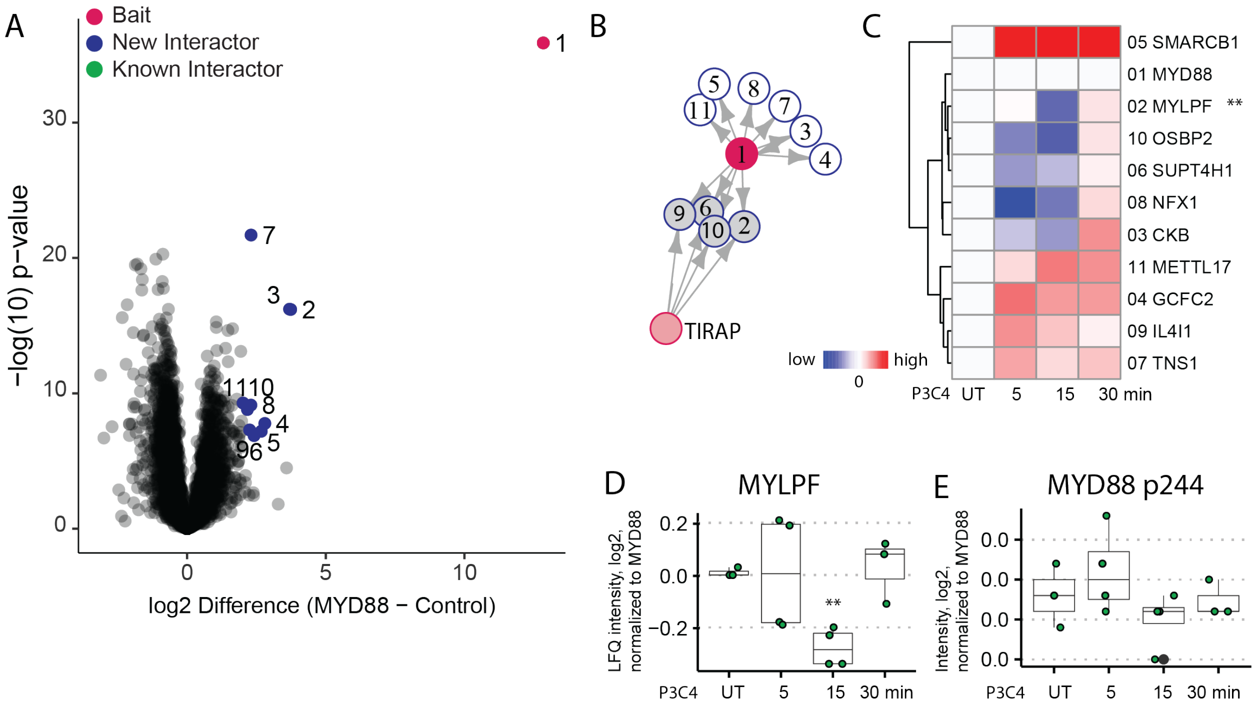

Appendix Figure S9. Related to [Fig. 4, Table 1,2,3]: Interactions and PTMs of MYD88 in Human Monocytes. (A) Volcano plot representing the interactome of MYD88 (measured 14x in biological replicates) compared against all other pull-downs in the control group. Significant interactors of MYD88 (two-tailed t-test, FDR < 0.01, enrichment >4) are colored in blue (novel interactors) and green (known interactors). (B) Interactors of MYD88 (blue: novel interactors, green: known interactors) with interconnecting proteins between different baits colored in grey. (C) Heatmap of significant interactors of MYD88 upon TLR2 activation of human monocytes, with significant hits (two-sided *t*-test, p-value < 0.05) denoted with an asterisk. Cell activation was performed for 5, 15 and 30 min with the TLR2 ligand PAM3CSK4 (P3C4), UT was harvested after 30 min without Pam3CSK4 activation (D) LFQ Intensity profile of MYD88 interactor MYLPF upon TLR2 activation of human monocytes, normalized to MYD88 bait LFQ intensity and untreated condition. (E) Intensity profile of phosphorylation of MYD88 position 244 upon TLR2 activation of human monocytes, normalized to MYD88 intensity and untreated condition. ***: p-value < 0.001, **: p-value < 0.01, *: p-value < 0.05).

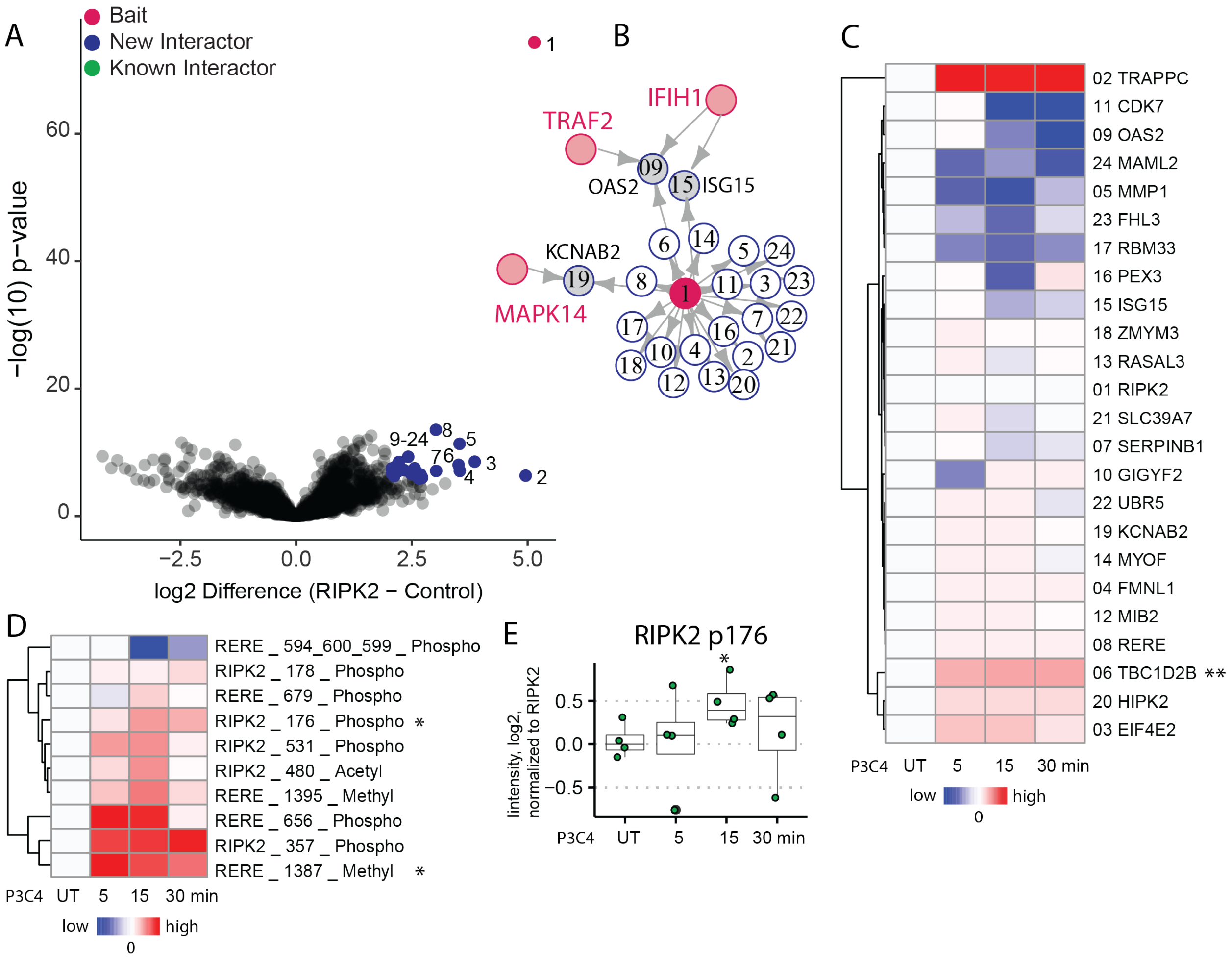

Appendix Figure S10. Related to [Fig. 4, Table 1,2,3]: Interactions and PTMs of RIPK2 in Human Monocytes. (A) Volcano plot representing the interactome of RIPK2 (measured 16x in biological replicates) compared against all other pull-downs in the control group. Significant interactors of RIPK2 (two-tailed t-test, FDR < 0.01, enrichment >4) are colored in blue (novel interactors) and green (known interactors). (B) Interactors of RIPK2 (blue: novel interactors, green: known interactors) with interconnecting proteins between different baits colored in grey. (C) Heatmap of significant interactors of RIPK2 upon TLR2 activation of human monocytes, with significant hits (two-sided *t*-test, p-value < 0.05) denoted with an asterisk. Cell activation was performed for 5, 15 and 30 min with the TLR2 ligand PAM3CSK4 (P3C4), UT was harvested after 30 min without Pam3CSK4 activation. (D) Heatmap of RIPK2 PTMs (acetylation, methylation, and phosphorylation) upon TLR2 activation of human monocytes, with significant hits (two-sided *t*-test, p-value < 0.05) denoted with an asterisk. (E) Intensity profile of RIPK2 p176 upon TLR2 activation of human monocytes, normalized to RIPK2 bait intensity and untreated condition. ***: p-value < 0.001, **: p-value < 0.01, *: p-value < 0.05).

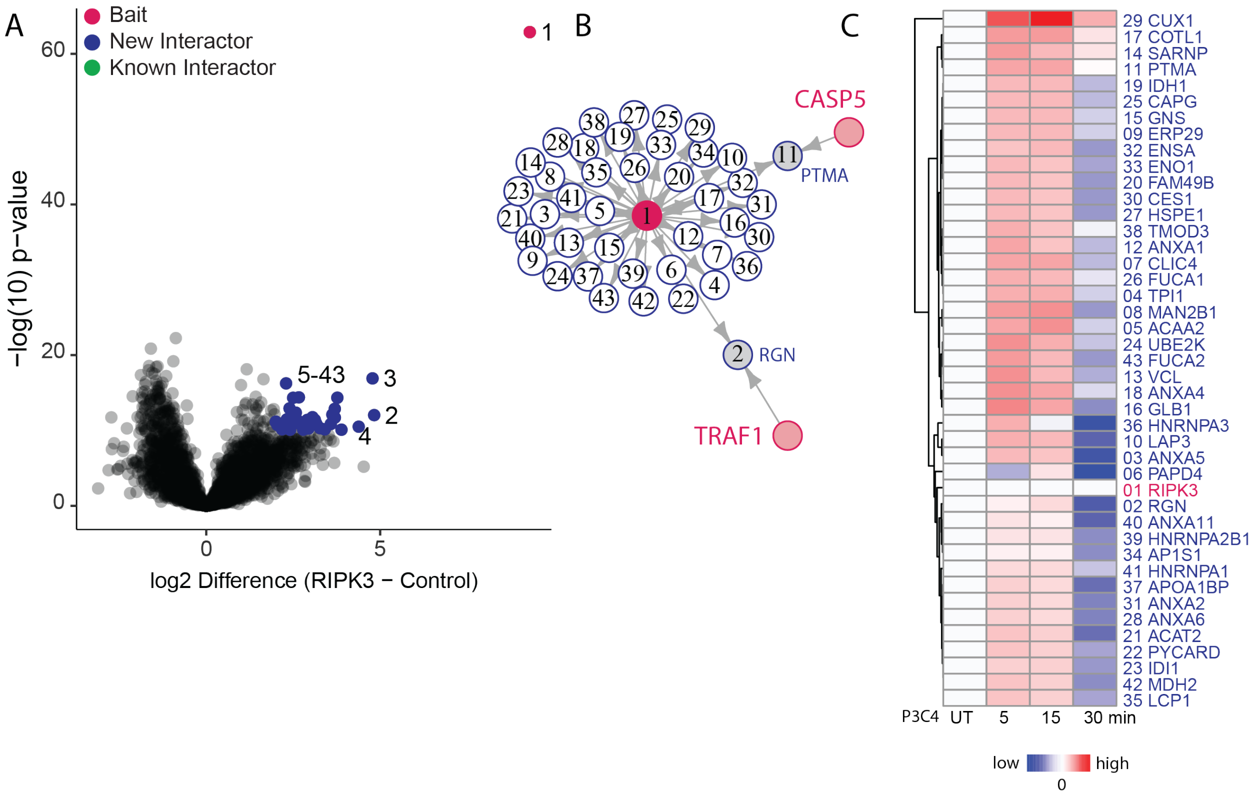

Appendix Figure S11. Related to [Fig. 4, Table 1,2,3]: Interactions and PTMs of RIPK3 in Human Monocytes. (A) Volcano plot representing the interactome of RIPK3 (measured 16x in biological replicates) compared against all other pull-downs in the control group. Significant interactors of RIPK3 (two-tailed t-test, FDR < 0.01, enrichment >4) are colored in blue (novel interactors) and green (known interactors). (B) Interactors of RIPK3 (blue: novel interactors, green: known interactors) with interconnecting proteins between different baits colored in grey. (C) Heatmap of significant interactors of RIPK3 upon TLR2 activation of human monocytes, with significant hits (two-sided *t*-test, p-value < 0.05) denoted with an asterisk. Cell activation was performed for 5, 15 and 30 min with the TLR2 ligand PAM3CSK4 (P3C4), UT was harvested after 30 min without Pam3CSK4 activation. ***: p-value < 0.001, **: p-value < 0.01, *: p-value < 0.05).

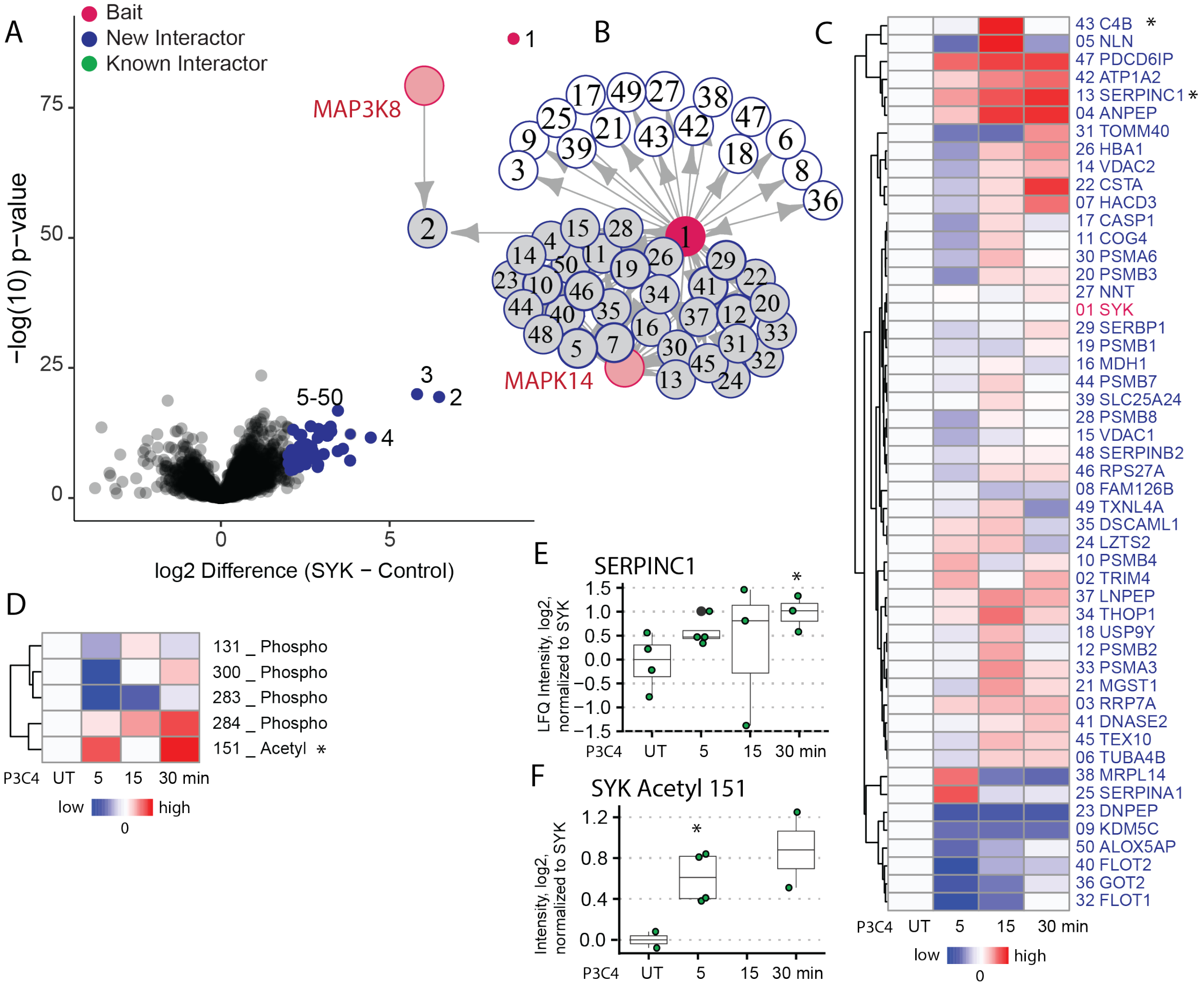

Appendix Figure S12. Related to [Fig. 4, Table 1,2,3]: Interactions and PTMs of SYK in Human Monocytes. *(A)* Volcano plot representing the interactome of SYK (measured 14x in biological replicates) compared against all other pull-downs in the control group. Significant interactors of SYK (two-tailed t-test, FDR < 0.01, enrichment >4) are colored in blue (novel interactors) and green (known interactors). (B) Interactors of SYK (blue: novel interactors, green: known interactors) with interconnecting proteins between different baits colored in grey. (C) Heatmap of significant interactors of SYK upon TLR2 activation of human monocytes, with significant hits (two-sided t-test, p-value < 0.05) denoted with an asterisk. Cell activation was performed for 5, 15 and 30 min with the TLR2 ligand PAM3CSK4 (P3C4), UT was harvested after 30 min without Pam3CSK4 activation. (D) Heatmap of SYK PTMs (acetylation, methylation, and phosphorylation) upon TLR2 activation of human monocytes, with significant hits (two-sided t-test, p-value < 0.05) denoted with an asterisk. (E) LFQ Intensity profile of SERPINC1 upon TLR2 activation of human monocytes, normalized to SYK bait LFQ intensity and untreated condition. (E) Intensity profile of SYK acetyl 151 upon TLR2 activation of human monocytes, normalized to SYK bait intensity and untreated condition. ***: p-value < 0.001, **: p-value < 0.01, *: p-value < 0.05).

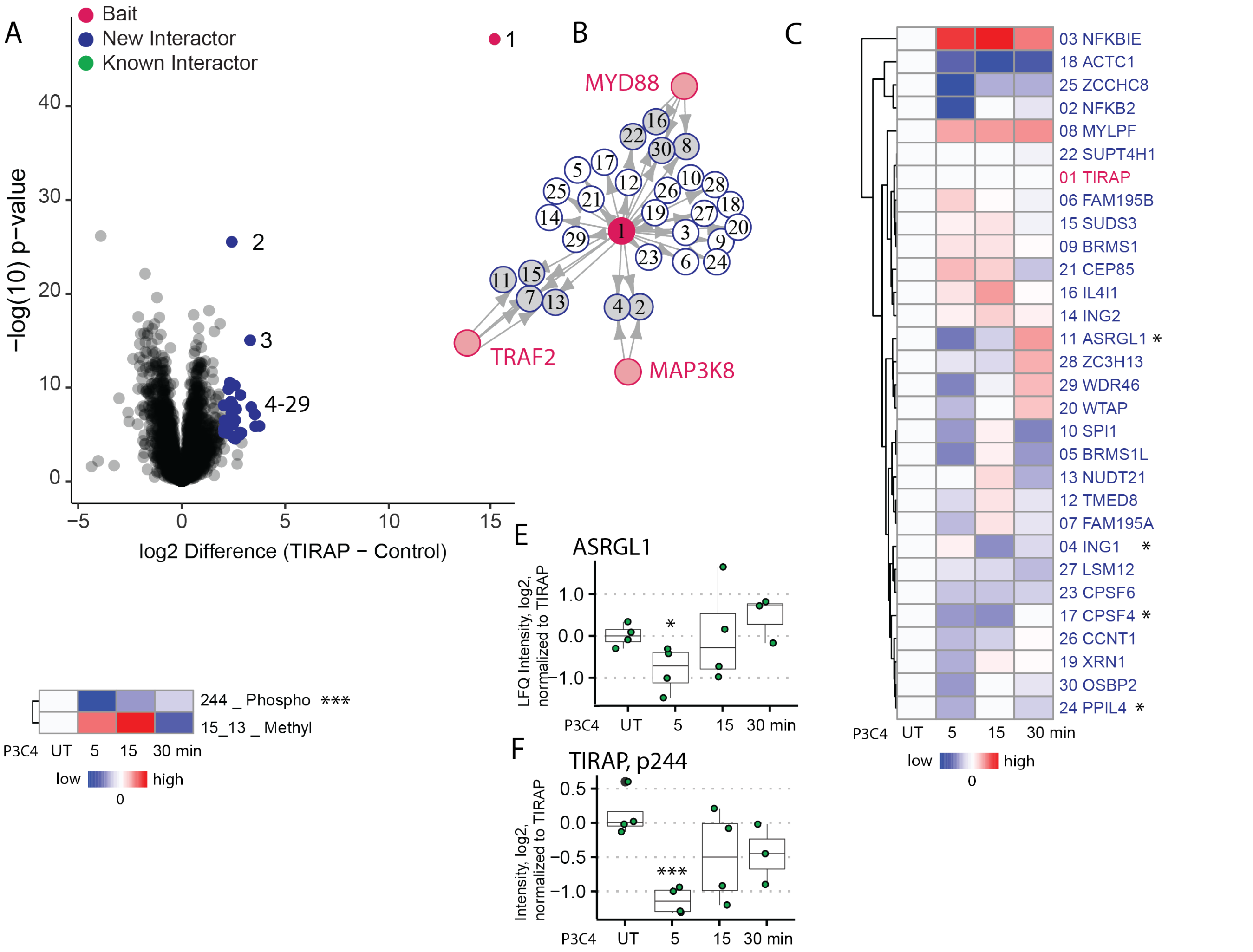

Appendix Figure S13. Related to [Fig. 4, Table 1,2,3]: Interactions and PTMs of TIRAP in Human Monocytes. (A) Volcano plot representing the interactome of TIRAP (measured 15x in biological replicates) compared against all other pull-downs in the control group. Significant interactors of TIRAP (two-tailed t-test, FDR < 0.01, enrichment >4) are colored in blue (novel interactors) and green (known interactors). (B) Interactors of TIRAP (blue: novel interactors, green: known interactors) with interconnecting proteins between different baits colored in grey. (C) Heatmap of significant interactors of TIRAP upon TLR2 activation of human monocytes, with significant hits (two-sided t-test, p-value < 0.05) denoted with an asterisk. Cell activation was performed for 5, 15 and 30 min with the TLR2 ligand PAM3CSK4 (P3C4), UT was harvested after 30 min without Pam3CSK4 activation. (D) Heatmap of TIRAP PTMs (acetylation, methylation, and phosphorylation) upon TLR2 activation of human monocytes, with significant hits (two-sided t-test, p-value < 0.05) denoted with an asterisk. (E) LFQ Intensity profile of ASRGL1 upon TLR2 activation of human monocytes, normalized to TIRAP bait LFQ intensity and untreated condition. (E) Intensity profile of TIRAP phospho 244 upon TLR2 activation of human monocytes, normalized to TIRAP bait intensity and untreated condition. ***: p-value < 0.001, **: p-value < 0.01, *: p-value < 0.05).

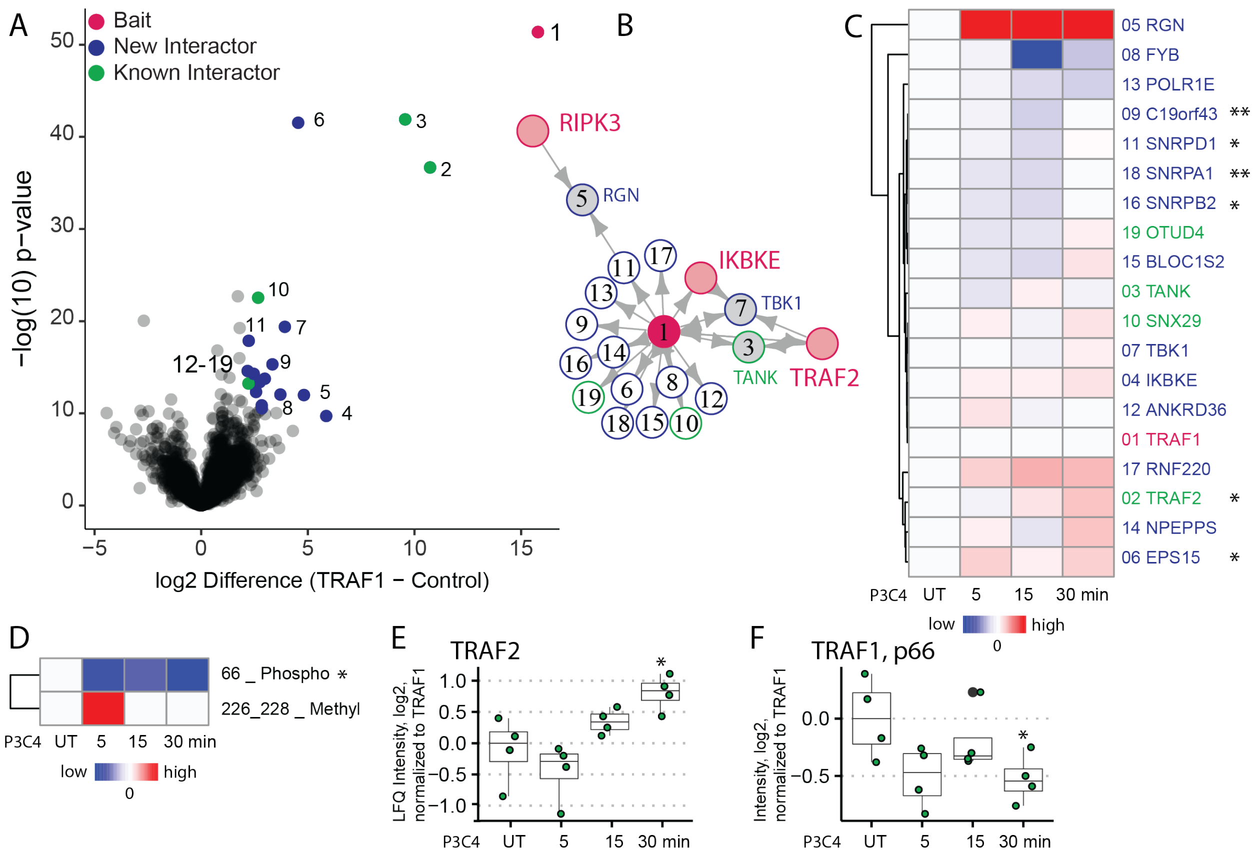

Appendix Figure S14. Related to [Fig. 4, Table 1,2,3]: Interactions and PTMs of TRAF1 in Human Monocytes. (A) Volcano plot representing the interactome of TRAF1 (measured 16x in biological replicates) compared against all other pull-downs in the control group. Significant interactors of TRAF1 (two-tailed t-test, FDR < 0.01, enrichment >4) are colored in blue (novel interactors) and green (known interactors). (B) Interactors of TRAF1 (blue: novel interactors, green: known interactors) with interconnecting proteins between different baits colored in grey. (C) Heatmap of significant interactors of TRAF1 upon TLR2 activation of human monocytes, with significant hits (two-sided t-test, p-value < 0.05) denoted with an asterisk. Cell activation was performed for 5, 15 and 30 min with the TLR2 ligand PAM3CSK4 (P3C4), UT was harvested after 30 min without Pam3CSK4 activation. (D) Heatmap of TRAF1 PTMs (acetylation, methylation, and phosphorylation) upon TLR2 activation of human monocytes, with significant hits (two-sided t-test, p-value < 0.05) denoted with an asterisk. (E) LFQ Intensity profile of TRAF2 upon TLR2 activation of human monocytes, normalized to TRAF1 bait LFQ intensity and untreated condition. (E) Intensity profile of TRAF1 phospho 66 upon TLR2 activation of human monocytes, normalized to TRAF1 bait intensity and untreated condition. ***: p-value < 0.001, **: p-value < 0.01, *: p-value < 0.05).

Appendix Table S1:

Name	Sequence (5'-->3')
Primer 1	ACCCAGCTTTCTTGTACCATCACCATCACCATCACCATCACCACCATGGTAGCGGCGACTACA AAGACGATGACGACAAGTAATGAACCGGGCGCGCCGCCCCTCTC
Primer 2	GTACAAGAAAGCTGGGTCCAAATTTAAATCATGGTGGAGCCTGCTTTTTTGTAC
Primer 3	AGGAATGCTCGTCAAGAA
Primer 4	GCCTGGAGACGCCATCCAC
Primer 5	TCCATATATGGGCTATGAACTAA
Primer 6	CTGCAGAATTCCACCAC
Primer 7	GTACAAAAAAGCAGGCTCC
Primer 8	GTACAAGAAAGCTGGGTCC
Primer 9	CTGCAGATATCACAAGTTTGTACA
Primer 10	CGCAAATGGGCGGTAGGCGTG

Appendix Table S2: Oligonucleotides for site directed mutagenesis of TRAF2

Name	Sequence (5'-->3')
Traf2 T7E fw	CATGGCTGCAGCTAGCGTGGAACCCCCTGGCTCCCTGGAG
Traf2 T7E rev	CTCCAGGGAGCCAGGGGGTTCCACGCTAGCTGCAGCCATG
Traf2 T7G fw	CATGGCTGCAGCTAGCGTGGGACCCCCTGGCTCCCTGGAG
Traf2 T7G rev	CTCCAGGGAGCCAGGGGGTCCCACGCTAGCTGCAGCCATG
Traf2 S11D fw	GACCCCCCCTGGCGATCTGGAGTTGCTAC
Traf2 S11D rev	GTAGCAACTCCAGATCGCCAGGGGGGGTC
Traf2 S11G fw	GTGACCCCCCCTGGCGGACTGGAGTTGCTACAG
Traf2 S11G rev	CTGTAGCAACTCCAGTCCGCCAGGGGGGGTCAC
K21R fw	gcccggcttctccagaaccctcctgggga
K21R rev	tccccaggagggttctggagaagccgggc
K27R fw	gtacttggcttccagtctggtccccaggagggt
K27R rev	accctcctggggaccagactggaagccaagtac
K31R fw	accaagctggaagccagatacctgtgctccgcc
K31R rev	ggcggagcacaggtatctggcttccagcttggt
K115R fw	gtattctttcagggtccctctccaggtgcatccatcact
K115R rev	agtgatggatgcacctggagagggaccctgaaagaatac
K119R fw	cagctctcgtattctctcagggtccccttcc
K119R rev	ggaaggggaccctgagagaatacgagagctg
K140R fw	cggaccaggcctctgcacgcgggac
K140R rev	gtcccgcgtgcagaggcctggtccg
K148R fw	ggtggcgctctctttcaccaaggcggaccag
K148R rev	ctggtccgccttggtgaaagagagcgccacc
K176R fw	cctcgtggtgcgctctcacgtctgctccg

K176R rev	cggagcagacgtgagagcgcaccacgagg
K184R fw	cacaagttaaggggaatctggggcagacctcgtgg
K184R rev	ccacgaggtctgccccagattccccttaacttgtg
K194R fw	cgggggatcttctttctgccgcagccgtcac
K194R rev	gtgacggctgcggcagaaagaagatcccccg
K195R fw	cccgggggatctttctcttgccgcagccg
K195R rev	cggctgcggcaagagaaagatcccccggg
K196R fw	ttctcccgggggattctcttcttgccgcagcc
K196R rev	ggctgcggcaagaagagaatcccccgggagaa
K201R fw	tgacgtggtcctgaaatctctcccgggggatcttc
K201R rev	gaagatcccccgggagagatttcaggaccacgtca
K207R fw	ctcgacacttgccacaagttctgacgtggtcctgaaacttc
K207R rev	gaagtttcaggaccacgtcagaacttgtggcaagtgtcgag
K211R fw	ctgcaagggactcgacatctgccacaagtcttgacgt
K211R rev	acgtcaagacttgtggcagatgtcgagtcccttgcag
K231R fw	cgtgctcctgctgtctctcaccctctacc
K231R rev	ggtagagggtgagagacagcaggagcacg-
K255R fw	ccaagaggggtcttgcctccagcaccgagctc
K255R rev	gagctcggtgctggaggcaagacccctcttgg
K277R fw	caaaagtggccgtctttctctccaggctctcgcac
K277R rev	gtgcgagagcctggagagaaagacggccacttttg
K278R fw	ttctcaaaagtggccgttctcttctccaggctctcgc
K278R rev	gcgagagcctggagaagagaacggccacttttgagaa
K313R fw	ctactcagggcttcaattctgtcttggtccagccggt
K313R rev	accggctggaccaagacagaattgaagccctgagtag
K320R fw	ctccagctgctgcactctgctactcagggcttc
K320R rev	gaagccctgagtagcagagtgcagcagctggag
K331R fw	gccatcgccaggtctctgaggccaatgctcc
K331R rev	ggagcattggcctcagagacctggcgatggc
K341R fw	cctccatctccaagactctctgctccaagtcagcc
K341R rev	ggctgacttggagcagagagtcttggagatggagg
K357R fw	cctggcgaagtctgagattctccagatgaagaccccatc
K357R rev	gatggggtcttcatctggagaatctcagacttcgccagg
K364R fw	tcctggcgcctcctggcgaagtctgag
K364R rev	ctcagacttcgccaggaggcgccagga
K389R fw	gatacgcagacacatcctgtagccgtacctgct
K389R rev	agcaggtacggctacaggatgtgtctgcgtatc
K415R fw	cattcgggcccctcatcaccacaaagaagaggg
K415R rev	ccctcttctttgtggtgatgaggggcccgaatg
K429R fw	taaggtcaccctctggttgaagggccaccg

K429R rev	cggtggcccttcaaccagagggtgacctta
K477R fw	tctttgcctccatcctggagacggggcag
K477R rev	ctgccccgtctccaggatggaggcaaaga
K481R fw	ccgcacgtaggaattccttgcctccatcttgga
K481R rev	tccaagatggaggcaaggaattcctacgtgcgg
K493R fw	ccacaatggccctgatgaagatggcatcgtccc
K493R rev	gggacgatgccatcttcatcagggccattgtgg

Appendix Table S3: Oligonucleotides for CRISPR-Cas9 gene knockout

Name	Sequence (5'-->3')
fw_non-target_1	CACCGGGGAGGTGGCTTTAGGTTTT
fw_non-target_2	CACCGACCACTAATGAGATTCTTGT
fw_non-target_3	CACCGGGGCAGAAGTTGCTGTCCTG
fw_non-target_4	CACCGTCAGCAAAGGACGAAACAAA
fw_non-target_5	CACCGGACAGTGAAATTAGCTCCCA
fw_non-target_6	CACCGCTTACCCCTATTATAATGAA
rev_non-target_1	AAACAAAACCTAAAGCCACCTCCCC
rev_non-target_2	AAACACAAGAATCTCATTAGTGGTC
rev_non-target_3	AAACCAGGACAGCAACTTCTGCCCC
rev_non-target_4	AAACTTTGTTTCGTCCTTTGCTGAC
rev_non-target_5	AAACTGGGAGCTAATTTCACTGTCC
rev_non-target_6	AAACTTCATTATAATAGGGGTAAGC
fw_Arhgef18_1	CACCGGACCATACCTTCTACAAAAA
fw_Arhgef18_2	CACCGGAGGCGGTGTAGGGATCTGA
fw_Arhgef18_3	CACCGGCAACTTCTCCATCGTGCGG
fw_Foxk2_1	CACCGGCTTTAGGATGCTTTGCGTG
fw_Foxk2_2	CACCGGTTCCCGTGGGGGAGGGCAG
fw_Foxk2_3	CACCGGCCGCACCTGATGGTTCCCG
fw_Fosb_1	CACCGGGGGTCTCCTAGGCCGGGCT
fw_Fosb_2	CACCGGCGGAACTACCAGTGGGCCT
fw_Fosb_3	CACCGGTCGACGACCGGGGGCTGGG
fw_Map3k7_1	CACCGGAGGAAAGCGTTTATTGTAG
fw_Map3k7_2	CACCGGAGTTGTTTGCAAAGCTAAG
fw_Map3k7_3	CACCGGTTTGTTTAACTCAGGTTGT
rev_Map3k7_3	AAACACAACCTGAGTTAAACAAACC
rev_Map3k7_2	AAACCTTAGCTTTGCAAACAACTCC
rev_Map3k7_1	AAACCTACAATAAACGCTTTCCTCC
rev_Fosb_3	AAACCCCAGCCCCCGGTCGTCGACC
rev_Fosb_2	AAACAGGCCCACTGGTAGTTCCGCC
rev_Fosb_1	AAACAGCCCGGCCTAGGAGACCCCC

rev_Foxk2_3	AAACCGGGAACCATCAGGTGCGGCC
rev_Foxk2_2	AAACCTGCCCTCCCCCACGGGAACC
rev_Foxk2_1	AAACCACGCAAAGCATCCTAAAGCC
rev_Arhgef18_3	AAACCCGCACGATGGAGAAGTTGCC
rev_Arhgef18_2	AAACTCAGATCCCTACACCGCCTCC
rev_Arhgef18_1	AAACTTTTTGTAGAAGGTATGGTCC

Appendix Table S4: Oligonucleotides for epitope tagging of MAPK14 with STREP-tag

Name	Sequence (5' --> 3')
MAPK14 forward	GTA CAA AAA AGC AGG CTC CAC CAT GTC TCA GGA GAG GC
MAPK14 reverse	GTA CAA GAA AGC TGG GTC CAA TCA TTT TTC AAA CTG CGG<br>ATG GCT CCA GGA CTC CAT CTC TTC TTG G

Appendix Table S5: Recombinant DNA from **Precision Lenti ORF Collection, GE Healthcare**

Name	Clone ID
AKT1	PLOHS_100067600
BCL10	PLOHS_ccsbBEn_02048
BIRC2	PLOHS_100010056
CASP4	PLOHS_100009633
CASP5	PLOHS_ccsbBEn_10709
CASP7	PLOHS_100066968
IFIH1	PLOHS_ccsbBEn_12450
MAP3K8	PLOHS_100072564
MAPK14	PLOHS_ccsbBEn_00371
MYD88	PLOHS_100067074
RIPK2	PLOHS_ccsbBEn_02010
RIPK3	PLOHS_ccsbBEn_11583
TIRAP	PLOHS_100008624
TRAF1	PLOHS_100071081
TRAF2	PLOHS_100009224
TRAF6	PLOHS_100008852

Appendix Table S6: Recombinant DNA by Gene synthesis

Name	Sequence (5' --> 3')
PGK weak promoter	CAATTGCGCGTCGTGCAGGACGTGACAAATGGAAGTAGCACGTCTCACTAGTCTCGTGCAGATGGACAGCACCGCTGAGCAATGGAAGCGGGTAGGCCTTTGGGGCAGCGGCCAATAGCAGCTTTGCTCCTTCGCTTTCTGGGCTCAGAGGCTGGGAAGGGGTGGGTCCGGGGGCGGGCTCA<br>GGGGCGGGCTCAGGGGCGGGGCGGGCGCCCGAAGGTCCTCCGGAGGCCCGGCATTCTGCACGCTTCAAAAGCGCACGTCTGCCGCGCTGTTCTCCTCTTCCTCATCTCCGGGCCTTTCGACCTGGCG
MAP3K7	GTACAAAAAAGCAGGCTCCACCATGTCTACAGCCTCTGCCGCCTCCTCCTCCTCCTCGTCTTCGGCCGGTGAGATGATCGAAGCCCCTTCCCAGGTCCTCAACTTTGAAGAGATCGACTACAAGGAGATCGAGGTGGAAGAGGTTGTTGGAAGAGGAGCCTTTGGAGTTGTTTGCAAAGCTAAG<br>TGGAGAGCAAAAGATGTTGCTATTAAACAAATAGAAAGTGAATCTGAGAGGAAAGCGTTTATTGTAGAGCTTCGGCAGTTATCCCGTGTGAACCATCCTAATATTGTAAAGCTTTATGGAGCCTGCTTGAATCCAGTGTGTCTTGTGATGGAATATGCTGAAGGGGGCTCTTTATATAATGTGC<br>TGCATGGTGCTGAACCATTGCCATATTATACTGCTGCCCACGCAATGAGTTGGTGTTTACAGTGTTCCCAAGGAGTGGCTTATCTTCACAGCATGCAACCCAAAGCGCTAATTCACAGGGACCTGAAACCACCAAACTTACTGCTGGTTGCAGGGGGGACAGTTCTAAAAATTTGTGATTTTGGT<br>ACAGCCTGTGACATTCAGACACACATGACCAATAACAAGGGGAGTGCTGCTTGGATGGCACCTGAAGTTTTTGAAGGTAGTAATTACAGTGAAAAATGTGACGTCTTCAGCTGGGGTATTATTCTTTGGGAAGTGATAACGCGTCGGAAACCCTTTGATGAGATTGGTGGCCCAGCTTTCCGAA<br>TCATGTGGGCTGTTCATAATGGTACTCGACCACCACTGATAAAAAATTTACCTAAGCCCATTGAGAGCCTGATGACTCGTTGTTGGTCTAAAGATCCTTCCCAGCGCCCTTCAATGGAGGAAATTGTGAAAATAATGACTCACTTGATGCGGTACTTTCCAGGAGCAGATGAGCCATTACAGTATCCTT<br>GTCAGTATTCAGATGAAGGACAGAGCAACTCTGCCACCAGTACAGGCTCATTCATGGACATTGCTTCTACAAATACGAGTAACAAAAGTGACACTAATATGGAGCAAGTTCCTGCCACAAATGATACTATTAAGCGCTTAGAATCAAAATTGTTGAAAAATCAGGCAAAGCAACAGAGTGAATCTGG<br>ACGTTTAAGCTTGGGAGCCTCCCGTGGGAGCAGTGTGGAGAGCTTGCCCCCAACCTCTGAGGGCAAGAGGATGAGTGCTGACATGTCTGAAATAGAAGCTAGGATCGCCGCAACCACAGGCAACGGACAGCCAAGACGTAGATCCATCCAAGACTTGACTGTAACTGGAACAGAACCTGGTCAGG<br>TGAGCAGTAGGTCATCCAGTCCCAGTGTCAGAATGATTACTACCTCAGGACCAACCTCAGAAAAGCCAACTCGAAGTCATCCATGGACCCCTGATGATTCCACAGATACCAATGGATCAGATAACTCCATCCCAATGGCTTATCTTACACTGGATCACCAACTACAGCCTCTAGCACCGTGCCC<br>AAACTCCAAAGAATCTATGGCAGTGTTTGAACAGCATTGTAAAATGGCACAAGAATATATGAAAGTTCAAACAGAAATTGCATTGTTATTACAGAGAAAGCAAGAACTAGTTGCAGAACTGGACCAGGATGAAAAGGACCAGCAAAATACATCTCGCCTGGTACAGGAACATAAAAAGCTTTT<br>AGATGAAAACAAAAGCCTTTCTACTTACTACCAGCAATGCAAAAAACAACTAGAGGTCATCAGAAGTCAGCAGCAGAAACGACAAGGCACTTCATTGGACCCAGCTTTCTTGTAC

IKBKE	ATGGAATACTGCAGCAGCGGCAGCCTGCTGAGCGTGCTGGAAAGCCCCGAGAATGCCTTCGGCCTGCCCGAGGATGAGTTCCTGGTGGTGCTGAGATGTGTGGTGGCCGGCATGAACCACCTGAGAGAAAACGGCATCGTGCACCGGGACATCAAGC<br>CCGGCAACATCATGCGGCTCGTGGGCGAGGAAGGCCAGAGCATCTACAAGCTGACCGACTTCGGAGCCGCCAGAGAGCTGGACGACGACGAGAAGTTCGTGTCCGTGTACGGCACCGAGGAATACCTGCACCCCGATATGTACGAGCGGGCCGTGCT<br>GAGGAAGCCCCAGCAGAAAGCTTTTGGCGTGACCGTGGACCTGTGGTCCATCGGCGTGACACTGTACCATGCCGCCACCGGCTCCCTGCCCTTCATCCCATTTGGCGGCCCTCGGCGGAACAAAGAAATCATGTACCGGATCACCACCGAGAAGCCTGCC<br>GGCGCTATTGCTGGCGCCCAGAGAAGAGAGAACGGCCCCCTGGAATGGTCCTACACCCTGCCCATCACCTGTCAGCTGAGCCTGGGCCTGCAGAGCCAGCTGGTGCCTATCCTGGCCAACATCCTGGAAGTGGAACAGGCCAAGTGTTGGGGCTTCGACC<br>AGTTCTTCGCCGAGACAAGCGACATCCTGCAGCGGGTGGTGGTGCACGTGTTCAGCCTGTCTCAGGCTGTGCTGCACCACATCTACATCCACGCCCACAACACAATCGCCATCTTCCAGGAAGCCGTGCACAAGCAGACCAGCGTGGCCCCCAGACACCAG<br>GAATATCTGTTCGAGGGCCACCTGTGTGTGCTGGAACCTAGCGTGTCCGCCCAGCACATTGCCCACACAACAGCCAGCAGCCCCCTGACCCTGTTCAGCACCGCCATCCCTAAGGGCCTGGCCTTCAGAGATCCCGCCCTGGACGTGCCCAAGTTCGTGCCT<br>AAAGTGGACCTGCAGGCCGACTACAACACCGCCAAAGGCGTGCTGGGAGCCGGCTATCAGGCTCTGAGACTGGCTAGAGCCCTGCTGGACGGCCAGGAACTGATGTTCAGAGGCCTGCACTGGGTCATGGAAGTGCTGCAGGCCACATGCCGGCGGACA<br>CTGGAAGTGGCCAGAACCAGCCTGCTGTACCTGAGCAGCAGCCTGGGCACCGAGAGATTCAGCTCTGTGGCCGGAACCCCCGAGATTCAGGAACTGAAGGCCGCTGCCGAGCTGCGGAGCAGACTGAGAACACTGGCCGAGGTGCTGAGCCGGTGCAGCC<br>AGAACATCACCGAGACACAGGAAAGCCTGTCCAGCCTGAACCGCGAGCTCGTGAAGTCCAGGGACCAGGTGCACGAGGACCGCAGCATCCAGCAGATCCAGTGCTGCCTGGACAAGATGAACTTTATCTATAAGCAGTTCAAGAAAAGCCGGATGCGCCCT<br>GGCCTGGGCTACAACGAGGAACAGATCCACAAGCTGGACAAAGTGAATTTCAGCCACCTGGCCAAGAGACTGCTGCAGGTGTTCCAGGAAGAGTGCGTGCAGAAGTACCAGGCCAGCCTCGTGACCCACGGCAAGCGGATGCGCGTGGTGCATGAGACACGGA<br>ATCACCTGAGGCTCGTGGGATGTAGCGTGGCCGCCTGTAATACCGAAGCCCAGGGCGTGCAGGAATCCCTGAGCAAGCTGCTGGAAGAACTGAGCCACCAGCTGCTGCAGGACAGAGCCAAAGGCGCTCAGGCCAGCCCTCCTCCTATCGCCCCTTACCCTAGCCCC<br>ACCCGGAAAGACCTGCTGCTGCACATGCAGGAACTGTGCGAGGGCATGAAGCTGCTGGCCTCCGACCTGCTGGACAACAACCGGATCATCGAGCGGCTGAACCGGGTGCCAGCTCCCCCTGATGTG
SYK	ATGGCCAGCAGCGGCATGGCTGACAGCGCCAACCACCTGCCCTTCTTTTTCGGCAACATCACCCGGGAGGAGGCAGAAGATTACCTGGTCCAGGGGGGCATGAGTGATGGGCTTTATTTGCTGCGCCAGAGCCGCAACTACCTGGGTGGCTTCGCCCTGTCCGTG<br>GCCCACGGGAGGAAGGCACACCACTACACCATCGAGCGGGAGCTGAATGGCACCTACGCCATCGCCGGTGGCAGGACCCATGCCAGCCCCGCCGACCTCTGCCACTACCACTCCCAGGAGTCTGATGGCCTGGTCTGCCTCCTCAAGAAGCCCTTCAACCGGCCCC<br>AAGGGGTGCAGCCCAAGACTGGGCCCTTTGAGGATTTGAAGGAAAACCTCATCAGGGAATATGTGAAGCAGACATGGAACCTGCAGGGTCAGGCTCTGGAGCAGGCCATCATCAGTCAGAAGCCTCAGCTGGAGAAGCTGATCGCTACCACAGCCCATGAAAAA<br>ATGCCTTGGTTCCATGGAAAAATCTCTCGGGAAGAATCTGAGCAAATTGTCCTGATAGGATCAAAGACAAATGGAAAGTTCCTGATCCGAGCCAGAGACAACAACGGCTCCTACGCCCTGTGCCTGCTGCACGAAGGGAAGGTGCTGCACTATCGCATCGACAAAG<br>ACAAGACAGGGAAGCTCTCCATCCCCGAGGGAAAGAAGTTCGACACGCTCTGGCAGCTAGTCGAGCATTATTCTTATAAAGCAGATGGTTTGTTAAGAGTTCTTACTGTCCCATGTCAAAAAATCGGCACACAGGGAAATGTTAATTTTGGAGGCCGTCCACAACTTCCAG<br>GTTCCCATCCTGCGTCCTCCCCTGCCCAAGGGAACCGGCAAGAGAGTACTGTGTCATTCAATCCGTATGAGCCAGAACTTGCACCCTGGGCTGCAGACAAAGGCCCCCAGAGAGAAGCCCTACCCATGGACACAGAGGTGTACGAGAGCCCCTACGCGGACCCCGAGGAGAT<br>CAGGCCCAAGGAGGTTTACCTGGACCGAAAGCTGCTGACGCTGGAAGACAAAGAACTGGGCTCTGGTAATTTTGGAACTGTGAAAAAGGCTACTACCAAATGAAAAAAGTTGTGAAAACCGTGGCTGTGAAAATACTGAAAAACGAGGCCAATGACCCCGCTCTTAAAG<br>ATGAGTTATTAGCAGAAGCAAATGTCATGCAGCAGCTGGACAACCCGTACATCGTGCGGATGATCGGGATATGCGAGGCCGAGTCCTGGATGCTGGTTATGGAGATGGCAGAACTTGGTCCCCTCAATAAGTATTTGCAGCAGAACAGACATGTCAAGGATAAGAACATCATAGAA<br>CTGGTTCATCAGGTTTCCATGGGCATGAAGTACTTGGAGGAGAGCAATTTTGTGCACAGAGATCTGGCTGCAAGAAATGTGTTGCTAGTTACCCAACATTACGCCAAGATCAGTGATTTCGGACTTTCCAAAGCACTGCGTGCTGATGAAAACTACTACAAGGCCCAGACCCATGGAAA<br>GTGGCCTGTCAAGTGGTACGCTCCGGAATGCATCAACTACTACAAGTTCTCCAGCAAAAGCGATGTCTGGAGCTTTGGAGTGTTGATGTGGGAAGCATTCTCCTATGGGCAGAAGCCATATCGAGGGATGAAAGGAAGTGAAGTCACCGCTATGTTAGAGAAAGGAGAGCGGATGGG<br>GTGCCCTGCAGGGTGTCCAAGAGAGATGTACGATCTCATGAATCTGTGCTGGACATACGATGTGGAAAACAGGCCCGGATTCGCAGCAGTGGAACTGCGGCTGCGCAATTACTACTATGACGTGGTGAACTAA
