# Supplementary material for: Identification of covalent modifications regulating immune signaling complex composition and phenotype
Source: Mol Syst Biol. 2021 Jul 28;17(7):e10125. doi: 10.15252/msb.202010125 (PMC8447602; doi:10.15252/msb.202010125)
Supplement: Supplementary file 3 — Expanded View Figures PDF [file MSB-17-e10125-s005.pdf]

Expanded View Figures

Figure EV1. Related to [Fig 1]: IMAP-MS in primary macrophages and T cells.

A Overview of MIP-APMS experiments performed to study TLR2 activation and drug perturbation of MAPK14 in human immune cells.
B Lentiviral transduction efficiency of pLOC-MAPK14 in U937 cells after antibiotic selection (2 d) determined by the GFP fluorescence of IRES-GFP. Images represent three replicate experiments. Bar represents median % GFP-positive cells from 3 experiments, error bars s.d.
C Workflow for IMAP-MS in primary human macrophages.
D Interactome of MAPK14 and MAP3K7 in primary human macrophages (measured in quadruplicates) compared against each other.
E Interactome of MAPK14 in primary human T4 cells (measured in quadruplicates) compared against each other.

 © 2021 The Authors

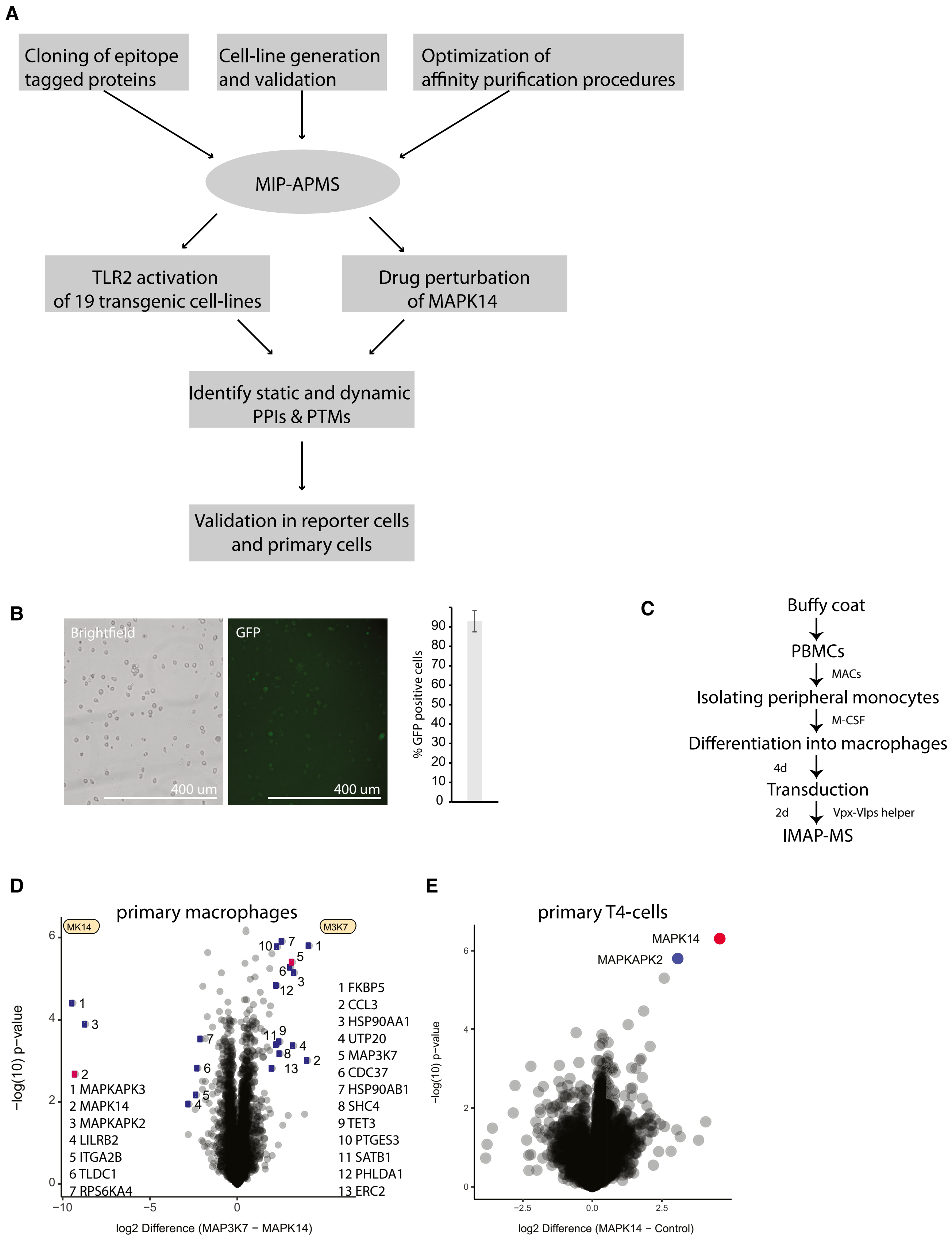
A
Cloning of epitope tagged proteins
Cell-line generation and validation
Optimization of affinity purification procedures
MIP-APMS
TLR2 activation of 19 transgenic cell-lines
Drug perturbation of MAPK14
Identify static and dynamic PPIs & PTMs
Validation in reporter cells and primary cells
B
Brightfield
GFP
400 um
400 um
% GFP positive cells
C
Buffy coat
PBMCs
MACs
Isolating peripheral monocytes
M-CSF
Differentiation into macrophages
4d
Transduction
2d
Vpx-Vlps helper
IMAP-MS
D
primary macrophages
MK14
M3K7
−log(10) p−value
1 MAPKAPK3
2 MAPK14
3 MAPKAPK2
4 LILRB2
5 ITGA2B
6 TLDC1
7 RPS6KA4
1 FKBP5
2 CCL3
3 HSP90AA1
4 UTP20
5 MAP3K7
6 CDC37
7 HSP90AB1
8 SHC4
9 TET3
10 PTGES3
11 SATB1
12 PHLDA1
13 ERC2
log2 Difference (MAP3K7 − MAPK14)
E
primary T4-cells
MAPK14
MAPKAPK2
−log(10) p−value
log2 Difference (MAPK14 − Control)

Figure EV1.

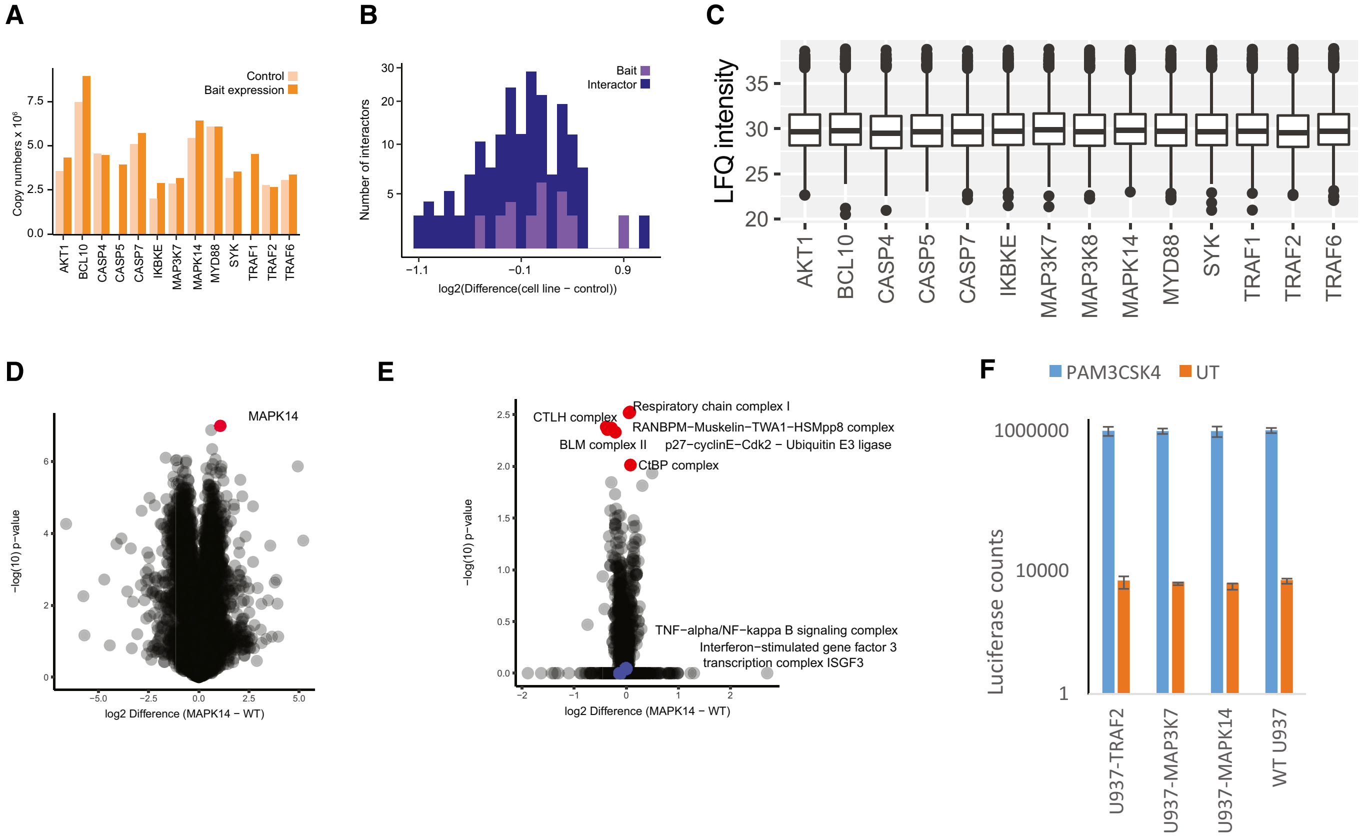

Figure EV2. Evaluation of Cell Line Properties used for IMAP-MS.

A Evaluation of the effect of bait overexpression on the proteomes of 13 bait cell lines (5,752 total protein IDs). Comparison of the copy numbers of overexpressed bait proteins in the non-overexpressing cells (U937 WT) and bait-overexpressing U937 cells. Copy numbers of bait proteins were calculated with the Perseus Plugin based on the LFQ intensity of the respective bait protein. LFQ intensities of endogenous protein and the overexpressed bait proteins are summarized.

B Evaluation of the effect of bait overexpression on interactor expression, plotted as prey expression difference between the control and bait-transduced cells.

C Total protein LFQ intensity in full proteomes across different cell lines. Central band of the boxplot shows the median, boxes represent the IQR, and 3 biological replicates were performed for every condition.

D Protein expression difference of wildtype U937 versus transduced U937 (MAPK14-U937; measured in triplicates, bait protein shown in red).

E 1D Annotation enrichment comparing U937 WT versus transduced U937 (MAPK14-U937) with significantly changing Corum annotations highlighted in red and infection-associated pathways highlighted in blue.

F Luciferase assay of wild-type U937 and transduced U937 cell lines (MAPK14-U937, MAP3K7-U937, TRAF2-U937) comparing raw intensity counts of non-activation (orange) versus PAM3CSK4 activation (blue). Bars represent median, error bars represent s.d., and four biological replicates were performed per condition.

    © 2021 The Authors

Figure EV3. Related to [Fig 1]: Comparison of His-IMAC, Anti-Flag, and Anti-Strep enrichment as well as evaluation of IMAP-MS bait properties.

A Interactome of MAPK14 with different IP strategies from HEK cells transfected with MAPK14-HisGSGFlag or MAPK14-Strep-tag (measured 3×, bait proteins are shown in red, prey proteins significant in His-IMAC in blue)

B Comparison of background proteins between His, Flag, and Strep beads. Proteins that were identified and quantified at least once were included in the Venn diagram.

C Sequence coverage of MAPK14 with different IP strategies from HEK cells transfected with MAPK14-HisGSGFlag or MAPK14-Strep-tag. Each bar represents the median of three experiments. Bars represent median, and error bars represent s.d.

D Protein intensity (log_2) of MAPK14 after enrichment with different affinity matrices. Each enrichment was performed with equal bead amounts (50 μL each) and 5 Mio cells as input. Each bar represents the median of three experiments. Bars represent median, and error bars represent s.d.

E, F Correlation of sequence coverage and bait protein intensity for different imidazole concentrations in loading buffer (E) and wash buffer (F) is shown. Dot size indicates protein purity (in %).

G Difference (log_2) of baits versus control for all identified proteins in each interactome (see also Table S1). Bait proteins are depicted in red and all other identified proteins in turquoise.

H Median intensities (log_2), peptide numbers, sequence coverage, and bait LFQ variation of bait proteins. For median bait LFQ variation, bait proteins were normalized to one of the replicates and median difference was plotted. Central band of the boxplot shows the median, boxes represent the IQR, and 19 independent replicates (i.e., 19 bait proteins) were used.

I Median Pearson correlation between pull-downs in different control groups. Central band of the boxplot shows the median, boxes represent the IQR, and 19 independent replicates (i.e., 19 bait proteins) were used.

J Comparison of different pull-downs reveals high correlation between LFQ intensities within three different background control groups.

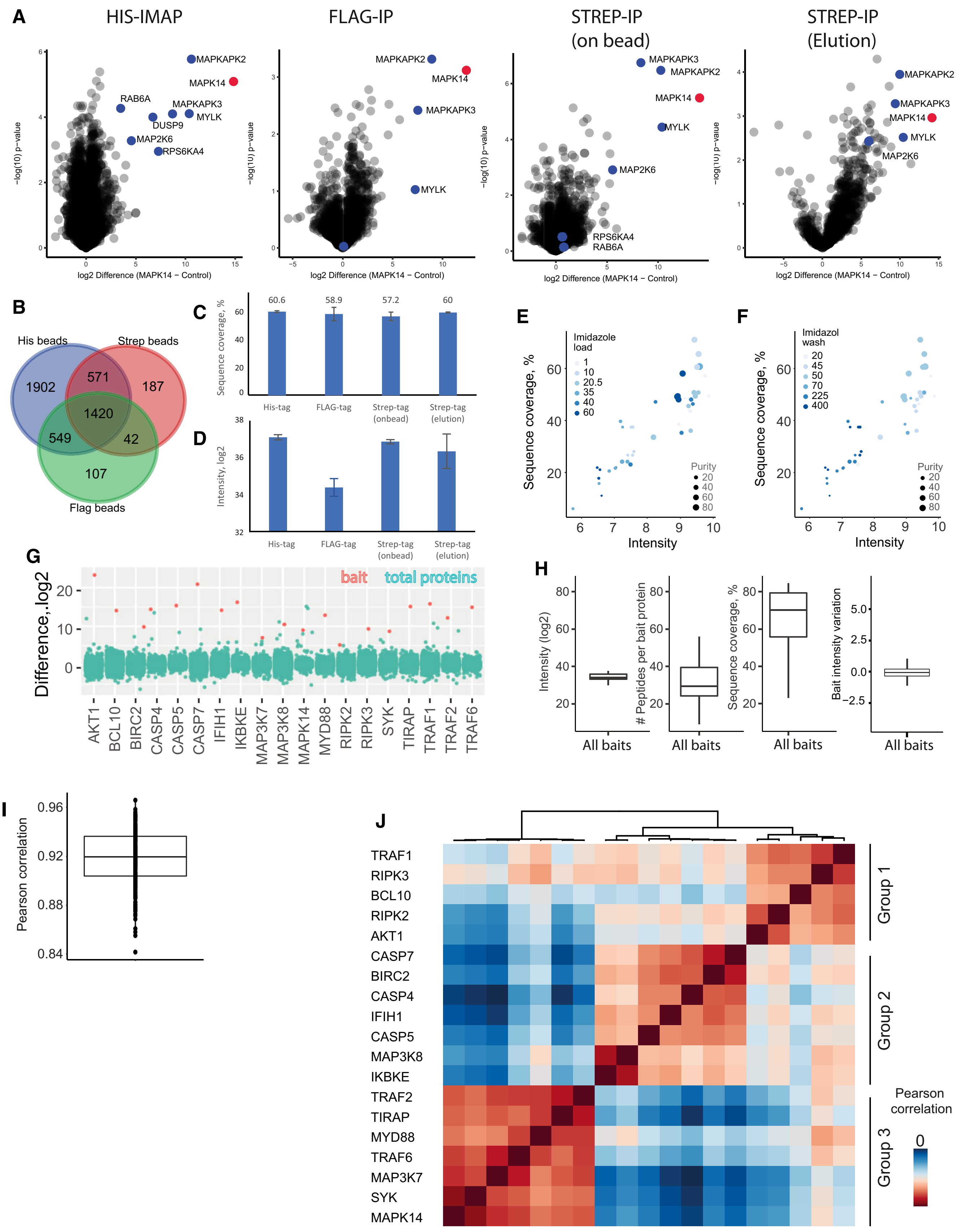

Figure EV3.

© 2021 The Authors

Figure EV4. Related to [Fig 2, Table 3]: Evaluation of cellular pathway activation and PTM dynamics.

A Pie chart showing the proportion of quantified bait/prey proteins (blue) in comparison with background proteins (red)

B Boxplots with enrichment differences ($\log_2$) of bait/prey versus background proteins. Central band of the boxplot shows the median, boxes represent the IQR, and 19 independent replicates (i.e., 19 bait proteins) were used. *P*-values were calculated by *t*-test. Asterisks indicate significant differences *****P*-value < 0.0001, **P*-value < 0.05, ****P*-value < 0.001.

C Boxplots with *P*-values ($-\log_{10}$) of bait/prey proteins versus background proteins. Central band of the boxplot shows the median, boxes represent the IQR, and 19 independent replicates (i.e., 19 bait proteins) were used. *P*-values were calculated by *t*-test. Asterisks indicate significant differences *****P*-value < 0.0001, **P*-value < 0.05, ****P*-value < 0.001.

D Impact of interconnected interactors on enrichment differences and *P*-values: Interactor calling was performed for indicated proteins using big (with SYK and MAPK14) and small (without SYK and MAPK14) control groups. Differences of all significant interactors and *P*-values are shown individually for SYK and MAPK14 pull-downs. Central band of the boxplot shows the median, boxes represent the IQR, and 16 biological replicates were used.

E Saint probability (probability of the interactor being a true interactor) plotted against the *P*-value from the Student's *t*-test comparing MAPK14 versus control (transduced with His-Tag only). Significant interactors from *t*-test analysis are colored in turquoise; SAINT interactors (75% probability of interactor being a true interactor) are above the horizontal line of 0.75. Known interactors are colored green.

F Heatmap with *z*-scored LFQ intensities of significantly interacting proteins for each bait protein (two-tailed *t*-test, FDR < 0.01, enrichment > 4) clustered by Euclidean distance. Baits are numbered as in Fig 2A.

G Number of modifications on MAPK14 detected with the indicated open search algorithms in at least one replicate. Open search was performed in MaxQuant (dependent peptide mode), MS Fragger, and Peaks/Taggraph on the drug mode of action dataset of MAPK14 (Fig 5). Mass offsets detected at distinct amino acid positions were kept separately.

H Venn diagram showing the overlap of modifications identified with open search algorithms in at least one replicate.

I Number of distinct modifications on MAPK14, identified in at least 70% of the replicates with MaxQuant (red), Peaks/Taggraph (violet), MaxQuant & Peaks/Taggraph (green) and with less than 70% valid values (turquoise) in all searches.

J Comparison of MaxQuant specific searches for MAP3K7, MAPK14, and TRAF2 to dependent peptide analysis (MaxQuant) and PEAKS/Taggraph. Identified and quantified sites are depicted in turquoise, not identified sites in red.

K Activation of wild-type and transgenic MAPK14-U937 cells with PAM3CSK4. Western blotting analysis using antibodies raised against phospho-MAPK14 and total MAPK14.

© 2021 The Authors

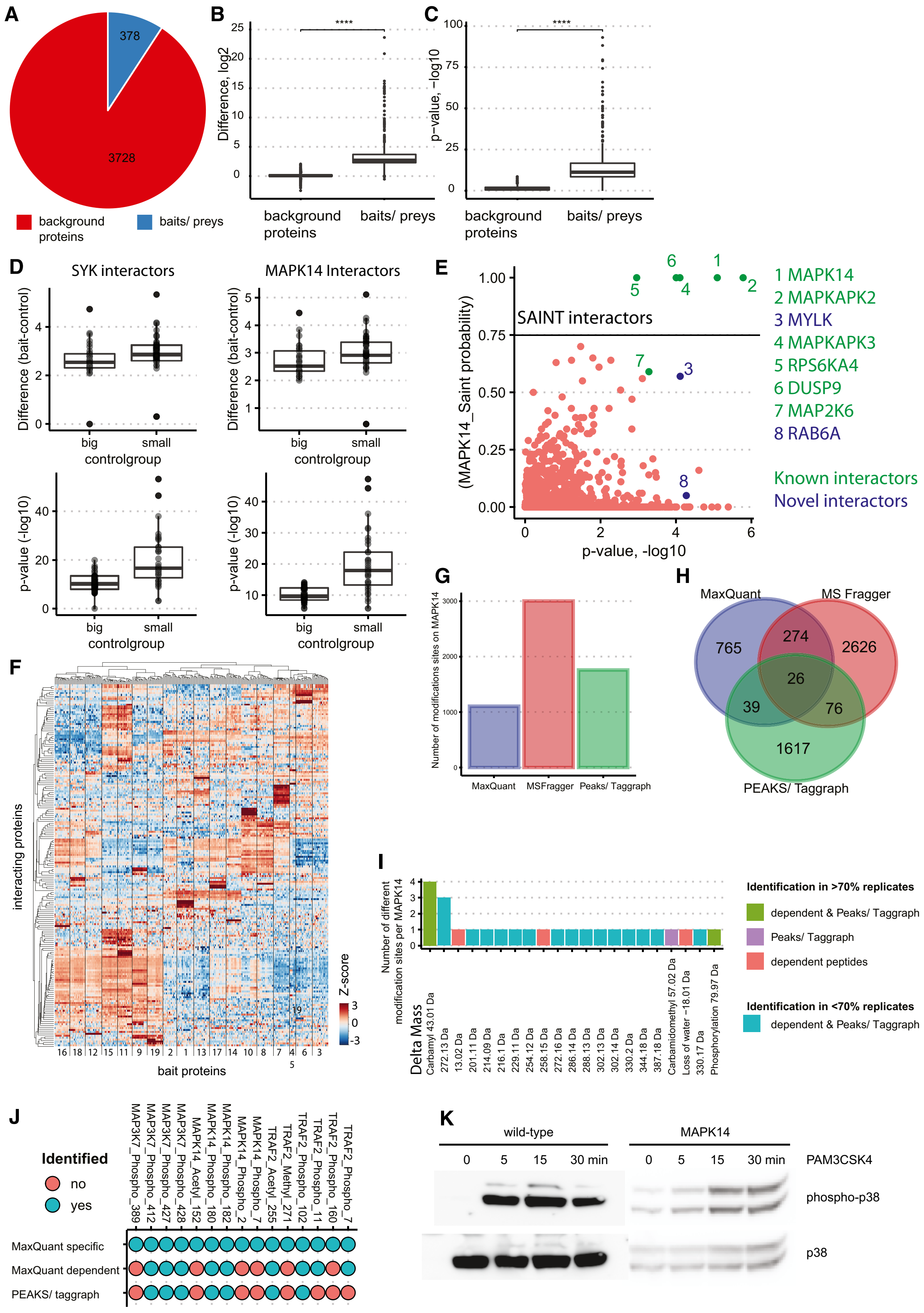

Figure EV4.

 © 2021 The Authors

Figure EV5. Related to [Fig 5, Table 5]: ISG15 expression in TRAF2 mutants and drug mode of action on MAP3K7 and TRAF2 analyzed to determine off-target effects.

A Overview of the dynamic and static PPIs and PTMs identified in 17 different cell lines.
B Global PPI and PTM differences upon PAM3CSK4 activation between prey proteins, and acetylated, methylated, and phosphorylated peptides.
C Comparison of TRAF2 interactors in native (0.05% NP-40 in lysis buffer) versus denaturing (6 M GdmCl in lysis buffer). Intensity profile of TRAF2 interactors in different TRAF2 phospho-variants, normalized to TRAF2 wild-type intensities.
D Differential expression of ISG15 in K->R TRAF2 mutants versus wild-type TRAF2 in full proteomes and interactomes. Central band of the boxplot shows the median, boxes represent the IQR, and 2 biological replicates were performed per condition.
E Analytical size-exclusion chromatography profile of TRAF2-ISG15 binding studies. For each analysis, the individual profiles are shown and indicated by color. Coomassie-stained SDS–PAGE lanes correlate with the approximate retention times in the chromatogram.
F Western blot analysis of CRISPR-KO reporter cell lines with antibodies raised against MAP3K7, ARHGEF18, FOSB. Tubulin was used as a loading control.
G Heatmap of MAP3K7 interactors after treatment with different MAPK14 inhibitors, with significant hits in at least one treatment (*t*-test, *P*–value < 0.05) denoted with an asterisk. Treatments were normalized to DMSO control.
H Heatmap of TRAF2 interactors after treatment with different MAPK14 inhibitors. Treatments were normalized to DMSO control.
I Intensity profile of MAP3K7 phosphorylation on Ser367, Ser412, and Ser445 after treatment with different MAPK14 inhibitors, normalized to MAP3K7 bait intensity. Central band of the boxplot shows the median, boxes represent the IQR, and four biological replicates were used per condition. *P*-values were calculated by *t*-test. Asterisks indicate significant differences ****P-value < 0.0001, *P-value < 0.05, ***P-value < 0.001.

© 2021 The Authors

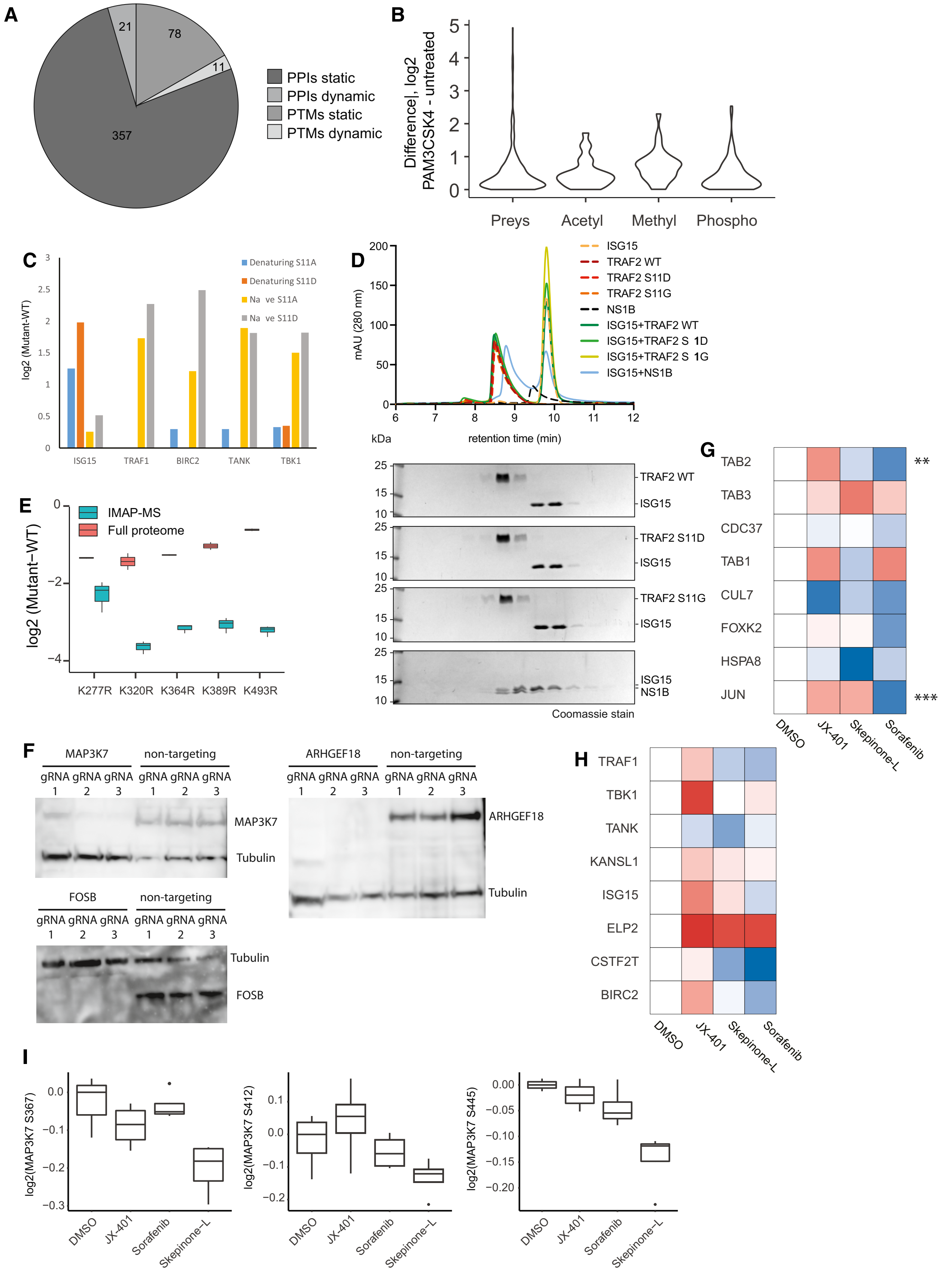

Figure EV5.

 © 2021 The Authors
