# Supplementary material for: Identification of covalent modifications regulating immune signaling complex composition and phenotype
Source: Mol Syst Biol. 2021 Jul 28;17(7):e10125. doi: 10.15252/msb.202010125 (PMC8447602; doi:10.15252/msb.202010125)
Supplement: Supplementary file 4 — Table EV1 [file MSB-17-e10125-s006.zip › Table EV1.docx]

**Table EV1:** Two-tailed T-test results for all baits. Interactomes of individual bait proteins are deposited in each tab with quantitative values and options for sorting by p-value (-log10) or median difference (log2) versus all other IPs. Column “Significant” shows the significant hits (permutation based FDR < 0.01 with 250 randomizations, enrichment > 2). In the columns “Bait (1-x)” LFQ-intensities of respective bait proteins and in the columns “Control (1-x)” LFQ-intensities of the controls are shown. Numbers were rounded to two digits. The tab “replicates” contains information about how many replicates per MIP-APMS experiment were performed. The tabe “Pride upload” contains information about raw files.
