# Supplementary material for: Identification of covalent modifications regulating immune signaling complex composition and phenotype
Source: Mol Syst Biol. 2021 Jul 28;17(7):e10125. doi: 10.15252/msb.202010125 (PMC8447602; doi:10.15252/msb.202010125)
Supplement: Supplementary file 5 — Table EV2 [file MSB-17-e10125-s004.zip › Table EV2.docx]

**Table EV2**: Dynamic interactors for all baits upon PAM3CSK4 activation. Interactors of individual bait proteins are deposited in each tab with quantitative values and options for sorting by p-value (-log10) or difference (log2) of different time-points of TLR2 activation versus untreated control. Column “Significant” shows the significant hits (p-value < 0.05) after Student’s T-test. Logarithmized LFQ intensities (in green) and normalized LFQ intensities (log2) to bait amount (in blue) as explained in materials and methods and to UT are shown for each replicate. Numbers were rounded to two digits.
