# Supplementary material for: Identification of covalent modifications regulating immune signaling complex composition and phenotype
Source: Mol Syst Biol. 2021 Jul 28;17(7):e10125. doi: 10.15252/msb.202010125 (PMC8447602; doi:10.15252/msb.202010125)
Supplement: Supplementary file 6 — Table EV3 [file MSB-17-e10125-s001.zip › Table EV3.docx]

**Table EV3:** Dynamic PTMs (Acetylation, Methylation and Phosphorylation) for all baits upon PAM3CSK4 activation. PTMs of individual bait proteins are deposited in each tab with quantitative values and options for sorting by p-value (-log10) or difference (log2) of different time-points of TLR2 activation versus untreated control. Column “Significant” shows the significant hits (p-value < 0.05) after Student’s T-test. Logarithmized peptide intensities (in green) and normalized logarithmized peptide intensities to bait amount (in blue) as explained in materials and methods and to UT are shown for each replicate. In the tab “Interdependency PPIs and PTMs” the results of the correlation analysis between PPIs and PTMs are displayed. PPIs and PTMs with 70% valid values in all replicates were considered. The LFQ intensities (PPIs) and peptide intensities (PTMs) normalized to bait input are displayed after z-scoring. The Cluster numbers are assigned as per Fig. 2G, H. Numbers were rounded to two digits.
