# Supplementary material for: Identification of covalent modifications regulating immune signaling complex composition and phenotype
Source: Mol Syst Biol. 2021 Jul 28;17(7):e10125. doi: 10.15252/msb.202010125 (PMC8447602; doi:10.15252/msb.202010125)
Supplement: Supplementary file 7 — Table EV4 [file MSB-17-e10125-s007.zip › Table EV4.docx]

**Table EV4:** Differential PPIs for different TRAF2 mutants (Phosphomutants and KR mutants for isgylation). Interactors of TRAF2 mutants (Phosphomutants, KR mutants, full-proteome KR mutants and KR mutants in primary cells) can be sorted by p-value (-log10) or difference (log2) of different mutants versus control. Column “Significant” shows the significant hits (p-value < 0.05) after Student’s T-test. Logarithmized LFQ intensities for different replicates and mutants are shown in blue. Normalized LFQ intensities of ISG15 to TRAF2 content in each sample are shown in green – they are additionally normalized to TRAF2-WT.
