# Supplementary material for: Identification of covalent modifications regulating immune signaling complex composition and phenotype
Source: Mol Syst Biol. 2021 Jul 28;17(7):e10125. doi: 10.15252/msb.202010125 (PMC8447602; doi:10.15252/msb.202010125)
Supplement: Supplementary file 8 — Table EV5 [file MSB-17-e10125-s008.zip › Table EV5.docx]

**Table EV5:** Dynamic PTMs and PPIs of MAPK14, MAP3K7 and TRAF2 upon small molecule treatment. Interactors and PTMs of MAPK14, MAP3K7 and TRAF2 upon small molecule treatment are deposited in each tab with quantitative values and options for sorting by p-value (-log10) or difference (log2) of small molecule treatment versus untreated control. Column “Significant” shows the significant hits (p-value < 0.05) after Student’s T-test.
